# Cyclooctatetraene-conjugated cyanine mitochondrial probes minimize phototoxicity in fluorescence and nanoscopic imaging†

**DOI:** 10.1039/d0sc02837a

**Published:** 2020-07-27

**Authors:** Zhongtian Yang, Liuju Li, Jing Ling, Tianyan Liu, Xiaoshuai Huang, Yuqing Ying, Yun Zhao, Yan Zhao, Kai Lei, Liangyi Chen, Zhixing Chen

**Affiliations:** Institute of Molecular Medicine, Beijing Key Laboratory of Cardiometabolic Molecular Medicine, Peking University Beijing China zhixingchen@pku.edu.cn lychen@pku.edu.cn; Peking-Tsinghua Center for Life Sciences, Peking University Beijing China; State Key Laboratory of Membrane Biology, Peking University Beijing China; Zhejiang Provincial Laboratory of Life Sciences and Biomedicine, Key Laboratory of Growth Regulation, Translational Research of Zhejiang ProvinceSchool of Life Sciences, Westlake University Hangzhou Zhejiang Province China leikai@westlake.edu.cn; Institute of Biology, Westlake Institute for Advanced Study Hangzhou Zhejiang Province China; PKU-Nanjing Institute of Translational Medicine Nanjing China

## Abstract

Modern fluorescence-imaging methods promise to unveil organelle dynamics in live cells. Phototoxicity, however, has become a prevailing issue when boosted illumination applies. Mitochondria are representative organelles whose research heavily relies on optical imaging, yet these membranous hubs of bioenergy are exceptionally vulnerable to photodamage. We report that cyclooctatetraene-conjugated cyanine dyes (PK Mito dyes), are ideal mitochondrial probes with remarkably low photodynamic damage for general use in fluorescence cytometry. In contrast, the nitrobenzene conjugate of Cy3 exhibits enhanced photostability but unaffected phototoxicity compared to parental Cy3. PK Mito Red, in conjunction with Hessian-structural illumination microscopy, enables 2000-frame time-lapse imaging with clearly resolvable crista structures, revealing rich mitochondrial dynamics. In a rigorous stem cell sorting and transplantation assay, PK Mito Red maximally retains the stemness of planarian neoblasts, exhibiting excellent multifaceted biocompatibility. Resonating with the ongoing theme of reducing photodamage using optical approaches, this work advocates the evaluation and minimization of phototoxicity when developing imaging probes.

## Abstract 2

Cyclooctatetraene-conjugated cyanine dyes represent an effective strategy to improve biocompatibility under light in live-cell fluorescence imaging and analysis of mitochondria.

## Graphical abstract

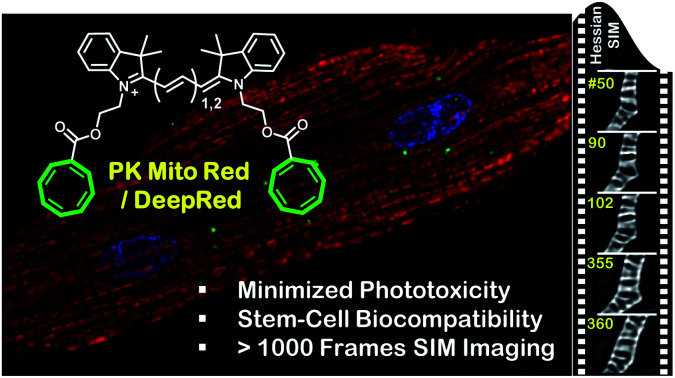

## Introduction

Mitochondria are crucial not only for ATP production, but also for other processes such as cell signaling and cell death.^1,2^ Mitochondria contain double-membrane structures that delicately compartmentalize biochemical transformations, dynamically interact with other cellular organelles, and adopt various shapes in different cells. To correlate mitochondrial function and metabolic states with their morphology and structure, modern mitochondrial research heavily relies on the development of novel live-cell imaging technologies. A representative example of this trend is the discovery of mitochondrial nanotunnels, whose dynamics were measured using live-cell fluorescence imaging in complement to the structural information observed with electron microscopy.^3^

**Fig. 1:**
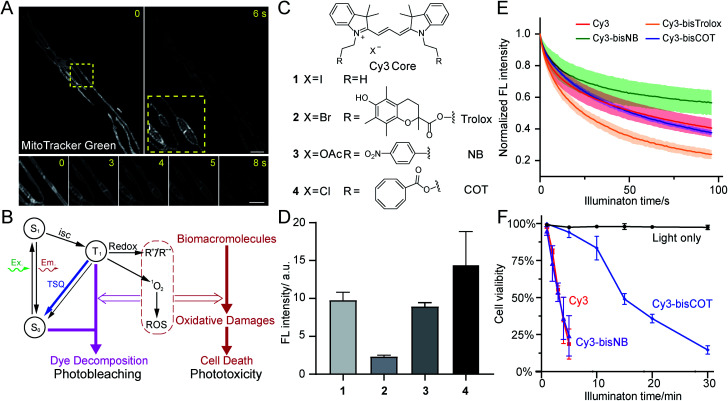
Evaluation of TSQ-conjugated mitochondrial dyes to alleviate phototoxicity. (A) Time-lapse images of human foreskin fibroblasts stained with MitoTracker Green FM and imaged by Hessian-SIM. Yellow boxes indicate the close-up view area. The image in the right yellow box is contrast-enhanced for better visualization of photo-induced swelling of mitochondria. (B) Diagram depicting the origin of photobleaching and phototoxicity. (C) Chemical structures of Cy3 core and TSQ-conjugated fluorophores. (D) Relative fluorescence intensities of HeLa cells stained with 250 nM compound **1–4** (averaged, *n* = 3). (E) Photobleaching of compound **1–4** in polymer films under confocal illuminations (averaged, *n* = 3). (F) Phototoxicity of compound **1**, **3**, **4** in HeLa cells (averaged, *n* = 3), measured by cell survival rate after green light illuminations (531/40 nm, 1.7 W cm^−2^). Scale bars: (A) 2 μm (upper) and 1 μm (lower).

Meanwhile, state-of-the-art, super-resolution (SR) microscopy has the potential to reveal intricate structures and novel dynamics of mitochondria in live cells in real time.^4^ SR imaging of mitochondria has been made possible by high-density, environmentally sensitive probes based on the single-molecule on-off switching principle.^5^ Recently, mitochondrial crista structures in live cells have been visualized with Hessian structured illumination microscopy (Hessian SIM)^6^ and stimulated emission depletion (STED) microscopy.^7–9^ Yet, in contrast to long-term SR imaging of other organelles, such as endoplasmic reticulum and the cytoskeleton, mitochondrial imaging is much more susceptible to phototoxicity. Extensive illumination induces rapid swelling of mitochondria, followed by the deformation and abruption of inner-membrane cristae (Fig. 1A). Ultimately, mitochondria transform into round, hollow structures. This process has been consistently observed with multiple types of SR microscopy,^7,10^ emerging as a major obstacle in preventing the visualization of physiologically relevant mitochondrial dynamics in live cells.

The growing need for better mitochondrial dyes makes it one of the most rapidly evolving fields in fluorescence indicator development,^11^ with several photostable scaffolds having been applied to mitochondrial imaging.^7,9,12,13^ Some of these dyes are sufficiently bright and stable to give strong signals even under STED microscopy. However, because the membranous structures of mitochondria are prone to the attack by the reactive oxygen species (ROS) associated with fluorescence excitation, focusing on improved photostability, alone, fails to maintain the health of mitochondria under prolonged light exposure.^7^ Moreover, because apoptosis can be triggered by the damaged mitochondria through ROS-related pathways,^10,14^ it is equally important to carefully evaluate the general toxicity of fluorescent probes beyond phototoxicity, particularly in the sorting of sensitive cells based on their metabolic states.

To improve mitochondrial analysis, we exploited a triplet-state engineering strategy to reduce the phototoxicity of cyanine-based mitochondrial dyes.^15^ This strategy was originally proposed to minimize photobleaching in dye lasers,^55^ single-molecule imaging and fluorescence resonance energy transfer (FRET) and has been applied to time-lapse imaging of protein targets in fixed and live cells.^16–19^ Blanchard and coworkers have further carried out researches showing decreased ROS generation of TSQ-conjugated dyes, suggesting applications towards reducing phototoxicity in biological conditions.^20,21^ We envision that this promising strategy, if proven compatible inside live cells, could benefit live-cell fluorescence analysis in general, such as in SR imaging and cytometry. We synthesized and evaluated a set of cyanine dyes conjugated with triplet-state quenchers (TSQ) and studied their cell localization and phototoxicity. Conjugation to cyclooctatetraene (COT), but not other tested anti-bleaching quenchers, reduced the phototoxicity of cyanines while strongly fluorescing in the mitochondria. This strategy has resulted in two selected molecules, PK Mito Red and PK Mito Deep Red, that fluoresce in the red and far red emission channels. PK Mito dyes outperform their commercial counterparts in viability during illumination of HeLa cells and isolated cardiomyocytes. Using these dyes, we achieved SR imaging with “crista clear” resolution for a record-long 2000 frames with minimal mitochondrial swelling, indicating that phototoxicity, rather than photostability, limits the duration of SR imaging of mitochondria. Finally, by transplanting PK Mito Red-loaded planarian stem cells into planarians devoid of endogenous SirNeoblasts, we demonstrated the minimal cellular toxicity of the dyes, highlighting their general potential for broad applications in live-cell mitochondrial analysis.

## Results

### Phototoxicity is a limiting factor of live-cell imaging

Phototoxicity is a universal phenomenon whose origin is commonly attributed to the high-energy species stemming from the excited states of chromophores (Fig. 1B). As triplet states are longer-lived than singlet states, they are often regarded as the primary source of phototoxicity. Usually the T_1_ state of dyes would sensitize molecular oxygen to singlet oxygen, which may further generate additional ROS species. Such reactive species could react with biomacromolecules and disrupt their structures and functions, producing phototoxicity. Membranous structures are particularly sensitive to photodynamic damage, as unsaturated lipids and proteins are susceptible to ROS attack. Notably, phototoxicity has long been harnessed for constructive applications, such as photodynamic therapy^22^ and chromophore-assisted light inactivation.^23^ At the same time, modern dyes tailored for live-cell imaging have been engineered to be minimally damaging. However, as fluorescence microscopy methods continuously evolve to break the diffraction barrier, live samples are inevitably subjected to increasing light intensities. Consequently, phototoxicity reemerged as a significant concern.^24^

The related process of photobleaching, which occurs when chromophores decompose, also warrants attention (Fig. 1B). Paradoxically, photobleaching receives disproportional attention compared to phototoxicity in SR imaging, although they both stem from triplet states. Selected chromophore scaffolds are elaborately tailored to withstand photo-decomposition; however, these strategies, in principle, do not necessarily translate to low phototoxicity. Moreover, in spite of advocates,^25^ phototoxicities of newly developed probes were not commonly measured and reported, giving few hints for further molecular engineering.

Because efficient assessments of phototoxicity are crucial, but highly customized for different sample types, we first established a reliable assay to quantify the phototoxicity of mitochondrial dyes.^5^ An epifluorescence microscope equipped with LED light cubes was used to provide continuous and even illumination over a large field of view covering hundreds of HeLa cells after a 40× objective (Fig. S1†). This illumination scheme provided light intensities comparable to those used in typical fluorescence imaging (up to 3 W cm^−2^). After exposing stained or unstained HeLa cells to relevant LED illumination for designated durations, the samples were allowed to recover for 2 h before treated with propidium iodide (PI). Subsequently, the illuminated cells were analyzed using a 10× objective. Cells with nuclear red fluorescence were counted as dead. This protocol efficiently measured nearly a hundred cells per run; therefore, reliably reporting cell viability after illumination.

We first evaluated the phototoxicities of excitation lights in the visible channels. While 1.7 W cm^−2^ green and 1.9 W cm^−2^ red LED lights exhibited negligible damage to HeLa cells, 2.5 W cm^−2^ blue LED light, itself, reduced cell viability in 10 min (Fig. S1C†). It is likely that the endogenous chromophores, particularly flavins and cytochromes, were excited by the blue light, resulting in ROS formation. This observation, corroborating previous studies,^25^ prompted us to develop probes excited by green and red light for minimal phototoxicity.

### COT is a privileged triplet-state quencher for cyanine dye in reducing mitochondrial phototoxicity

Having narrowed our design to red emission channels, cyanine dyes appeared to be ideal candidates for low-phototoxicity probes for several reasons. First, they generally have very high molecular extinction coefficients (>10^5^ M^−1^ cm^−1^) and reasonably high quantum yields (>10%), assuring high fluorescence signal.^26^ Second, red- and far-red-emitting carbocyanines (Cy3 and Cy5) are accessible through straightforward chemistry. In contrast, rhodamine and BODIPY dyes, while comparably bright, require more demanding synthetic chemistry (particularly their far-red emitting derivatives).^27^ Other dyes may have niche applications in bioimaging; however, most of them are excited by blue lights, and they generally have lower extinction coefficients. Moreover, lipophilic, positively charged carbocyanines tend to naturally accumulate inside or onto the mitochondrial inner membrane, where bears the lowest electrochemical potential in cells.^28^ Therefore, we selected two well-studied carbocyanine dyes, Cy3 and Cy5, for subsequent engineering of photocompatibility.

Because phototoxicity mainly results from the triplet states of excited fluorophores, we chose to exploit strategies that depopulate the triplet states to improve upon the cyanine-based family of mitochondrial dyes.^15^ Excited triplet states are harmful species that can either sensitize molecular oxygen to singlet oxygen or form highly reactive radicals that collectively account for the majority of the phototoxicity (Fig. 1B). The triplet states of dyes, however, can be depleted using triplet-state quenchers. This depletion process is drastically accelerated when the dye and quencher(s) are covalently conjugated; these bifunctional molecules are termed “self-healing dyes”.^15,17,18^ This strategy, pioneered by Blanchard and coworkers, has been increasingly applied to minimize photobleaching in single-molecule FRET studies.^29^ The Blanchard lab further studied the triplet-state quenching process of COT-conjugated cyanines^30^ and measured their triplet-state lifetime^17,30^ as well as relative reduction of ROS generation *in vitro*.^20^ These findings suggested the potential utility of self-healing dyes in alleviating phototoxicity in biological conditions. Despite the growing demand on reducing photodamage in the field of live-cell imaging, few further attempts along this direction were reported. Therefore, the cellular compatibility of TSQ conjugated fluorophores, such as their permeability and toxicity, demands detailed assessments.

Three popular TSQs (Trolox, nitrobenzene (NB), and COT) were conjugated to Cy3. Because the attachment of two TSQs with linkers of 2 or 3 chemical bonds has been suggested to optimally enhance photostability,^17^ the Cy3 derivatives were designed accordingly and synthesized using modular chemistry (Fig. 1C, Compounds **2**, **3**, and **4**, see ESI for details†). The resulting molecules – Cy3-bisTrolox (**2**), Cy3-bisNB (**3**), Cy3-bisCOT (**4**) – along with commercially available ethyl-derived Cy3 (**1**) were first incubated with HeLa cells (250 nM for 10 min) to assess their mitochondrial localization and fluorescence intensities. All of the four compounds exhibited characteristic mitochondrial distributions in live HeLa cells (Fig. S2†). However, the mitochondria of Cy3-bisTrolox-stained cells were significantly dimmer than those stained with the other three dyes (Fig. 1D). We attributed this result to the differences in the quenchers' molecular weight and hydrophobicity, which gave rise to diverse permeability of the dye derivatives. We then measured the photobleaching of the four molecules embedded in polyvinyl alcohol film under a confocal microscope. Cy3-bisCOT (**4**) and Cy3-bisNB (**3**) exhibited the lowest initial bleaching rates, while Cy3-bisTrolox (**2**) bleached the fastest (Fig. 1E). These data narrowed our selection to Cy3-bisCOT and Cy3-bisNB. Finally, these two dyes and the parental Cy3 molecule were subjected to our phototoxicity assay. Cy3-bisNB (**3**) and Cy3 (**1**) were equally phototoxic and killed all illuminated HeLa cells in 5 min, while Cy3-bisCOT (**4**) had drastically reduced cellular apoptosis with a half-lethal dose of 15 min (Fig. 1F). These results suggested that COT, but not NB, reduces the phototoxicity of Cy3 in mitochondria.

### PKMR and PKMDR outperform MitoTracker dyes in photo-compatibility

**Fig. 2:**
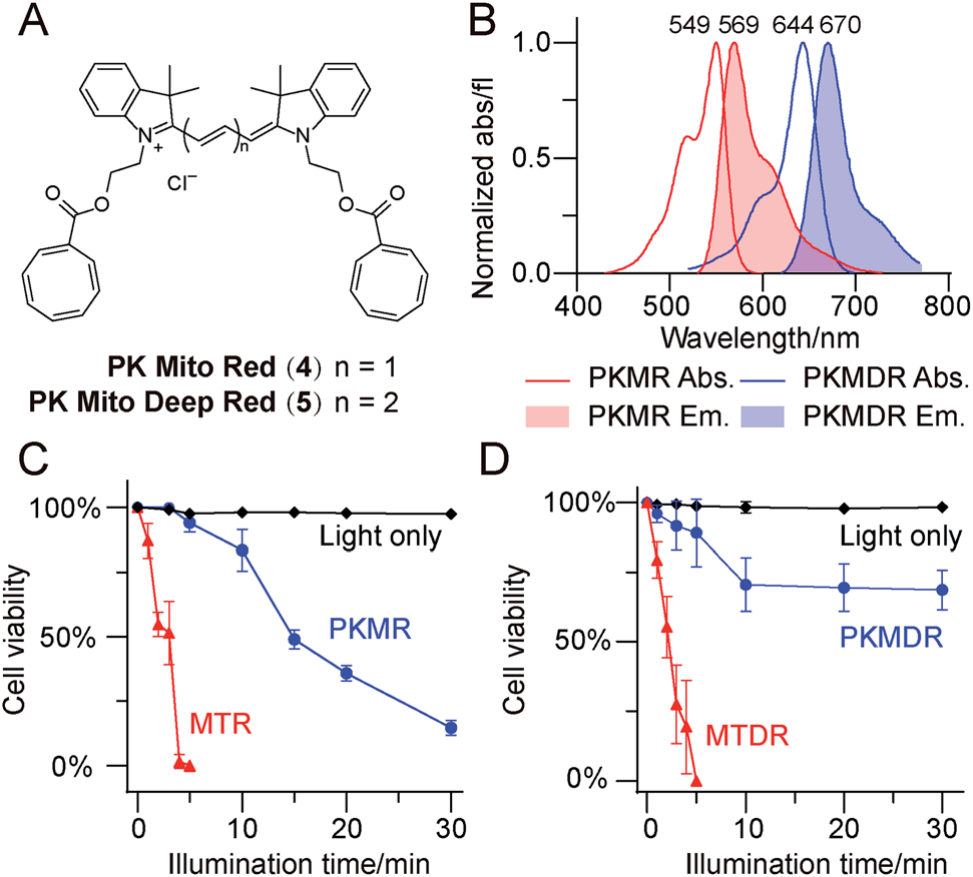
COT-conjugated mitochondrial dyes exhibit minimal phototoxicities in HeLa cells. (A) Chemical structures of PKMR and PKMDR. (B) Absorbance and emission spectra of PKMR and PKMDR in methanol. (C) Phototoxicity of PKMR and MTR CMXRos in HeLa cells (averaged, *n* = 3), measured by cell survival rate after green light illuminations (531/40 nm, 1.7 W cm^−2^). (D) Phototoxicity of PKMDR and MTDR in HeLa cells (averaged, *n* = 3), measured by cell survival rate after red light illuminations (628/40 nm, 1.9 W cm^−2^).

Having identified Cy3-bisCOT as a mitochondrial probe with minimal phototoxicity, we prepared the far-red emitting Cy5-bisCOT using similar chemistry (Fig. 2A and ESI,† chemical synthesis). It should be noted that the convenient and modular chemistry of these dyes, which required only 6 steps from commercial materials, ensured preparation of large quantities to assess their properties. The two dyes, Cy3-bisCOT (**4**) and Cy5-bisCOT (**5**), were named PK Mito Red (PKMR) and PK Mito Deep Red (PKMDR). Absorption/emission maxima in methanol were 549/569 nm for PK Mito Red and 644/670 nm for PK Mito Deep Red (Fig. 2B). The absorption and emission profiles were consistent in different solvents (Table S1†), rendering the dyes suitable for use with typical fluorescence microscopy and flow cytometry channels. Both dyes were readily loaded into HeLa cells in 15 min using a 250 nM staining concentration and resulted in patterns resembling mitochondria that co-localized with MitoTracker Green FM (Fig. S3†). Consistent with typical cationic mitochondrial probes, the accumulation of PK Mito dyes was dependent on the inner membrane potential, as pre-treatment with 20 μM of the oxidative phosphorylation uncoupler carbonyl cyanide 3-chlorophenylhydrazone reduced both overall dye fluorescence and mitochondrial specificity (Fig. S4†). The dark toxicities of the dyes in HeLa cells were evaluated using MTT assays. Comparable to the commercial MitoTracker Red CMXRos (MTR) and MitoTracker Deep Red FM (MTDR), the PK Mito dyes compromised cell viability by less than 30% after 12 h of continuous incubation with concentrations less than 2 μM (Fig. S5†). It should be noted that the long-term treatment with dyes for the MTT assays was harsher than the transient staining protocol routinely applied for imaging and flow cytometric analysis. These results indicated that the PK Mito dyes were suitable candidates for mitochondrial analysis with the desired fluorescence signal, good localization, and minimal toxicity.

We further compared the phototoxicity of PK Mito dyes with MTR/MTDR using our developed assay. MTR and MTDR killed all the HeLa cells after 5 min of illumination, with half-lethal doses of 2–3 min (Fig. 2C and D). Given that the illumination of cells without dyes resulted in no toxicity at the tested exposure durations (Fig. S5†), the observed cell death is unambiguously attributed to the phototoxicity of the MitoTracker dyes. PK Mito dyes, in contrast, showed significantly less cell death under photodamage. PK Mito Red had a half-lethal dose of 15 min light exposure, while PK Mito Deep Red exhibited a plateau in cell viability of >60%. These results established the remarkably low phototoxicity of COT-conjugated PK Mito dyes covering red and far red channels.

### Cardiomyocytes are well-preserved when imaged with PK Mito dyes

**Fig. 3:**
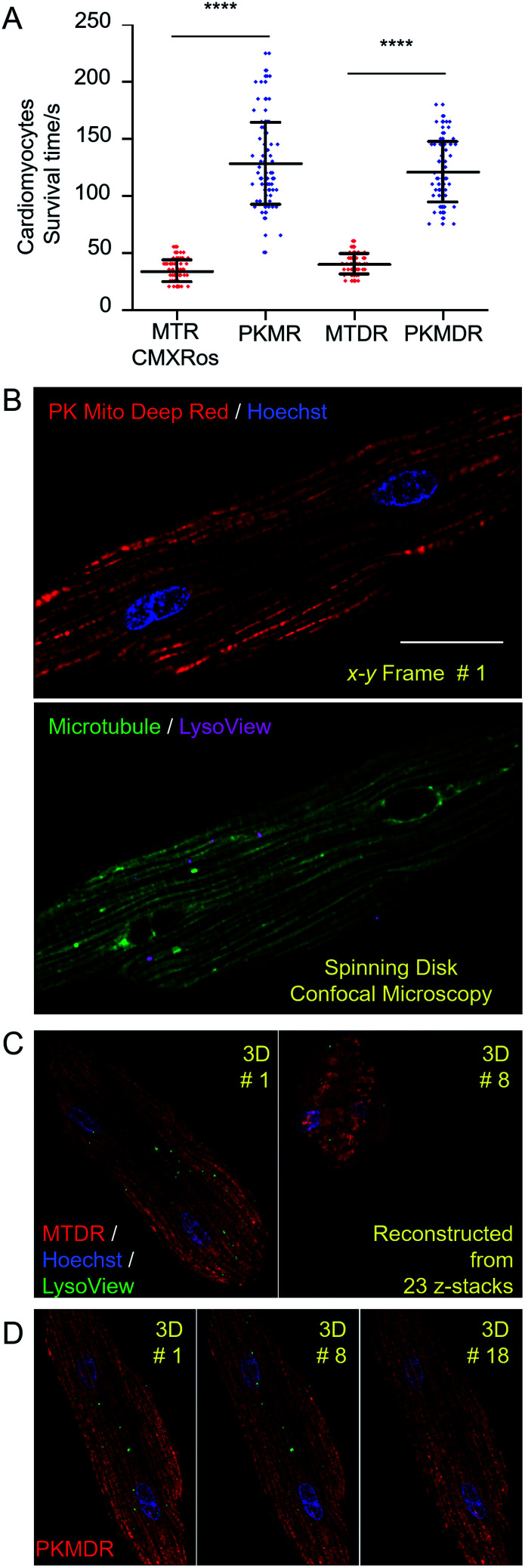
Mitochondrial imaging in live cardiomyocytes. (A) Phototoxicity of mitochondrial dyes in rat cardiomyocytes as measured by cell survival time under continuous laser illumination (1.4 W cm^−2^ 568 nm laser for red dyes and 1.2 W cm^−2^ 633 nm laser for deep red dyes). (B) Four-color 2D optical section images highlighting mitochondria, nuclei, microtubules, and lysosomes in an adult rat cardiomyocyte labeled with PK Mito Deep Red, Hoechst, ViaFluor 488 Live Cell Microtubule Stain, and LysoView 540. (C and D) Time-lapse 3D imaging of mitochondria, nuclei, and lysosomes labeled with MitoTracker Deep Red FM (C) and PK Mito Deep Red (D), 1 fps. Cell collapse occurred during the eighth imaging volume of the MTDR-labeled cardiomyocyte (C), while the PK Mito Deep Red-labeled cardiomyocyte remained morphologically intact for 18 imaging volumes (D). Scale bars: (B) 20 μm.

In order to establish the generality of PK Mito dyes in cell biology, we tested the compatibility of new dyes on isolated adult rat cardiomyocytes. Cardiomyocytes are one of the most metabolically active cells with a highest mitochondrial content. As a consequence, cardiomyocytes were more vulnerable to phototoxicity and exhibited a characteristic irreversible contraction behavior after excessive illuminations, representing a stringent evaluation on cell compatibility. The morphological change during photodynamic damage facilitated us to use an automated high-content imager to directly visualize the photo-toxification process. While cardiomyocytes stained with MTR rapidly collapsed after 34 ± 10 s of continuous illumination (1.4 W cm^−2^, 568 nm laser), and MTDR-stained cardiomyocytes collapsed after 40 ± 9 s of continuous illumination (1.2 W cm^−2^, 633 nm laser), the PK Mito dyes resulted in significantly longer observation time windows (130 ± 39 and 121 ± 27 s) under the same illumination power (Fig. 3A and S6†). It should be noted that the overall light doses to kill cardiomyocytes were consistently lower than those in triggering apoptosis in HeLa cells, underscoring the necessity of using PK Mito dyes.

To further demonstrate time-lapse imaging of mitochondria in cardiomyocytes, spinning disk confocal microscope was an ideal instrument due to its high imaging speed and gentle illumination. PK Mito Deep Red could be excited by low-energy red laser, which readily combined with chemical probes of DNA (Hoechst), microtubules (ViaFluor 488), and lysosomes (LysoView 540), and enabled four-color 3D imaging of live cardiomyocytes (Fig. 3B and Movie S1†). Time-lapse z-stacked imaging of a whole cell requires cumulative light exposure over both space and time, inducing considerable phototoxicity. In xyzt mode, cardiomyocytes stained with MTDR typically collapsed after 5–8 3D frames (Fig. 3C). In contrast, PK Mito Deep Red enabled 20-frame imaging composed of 23 z-stacked images before the single cardiomyocyte irreversibly contracted (Fig. 3D). It is noteworthy that most of the mitochondria were still bright at the time of cell contraction. This finding recapitulated the challenge of live-cell mitochondrial imaging, which is often not limited by the photobleaching of the mitochondrial markers, but by the accumulated photodamage. The PK Mito dyes thus offer a viable option to ameliorate the phototoxicity during time-lapse image acquisitions, particularly on vulnerable cardiomyocytes.

### PK Mito Red enables 2000-frame Hessian-SIM imaging

**Fig. 4:**
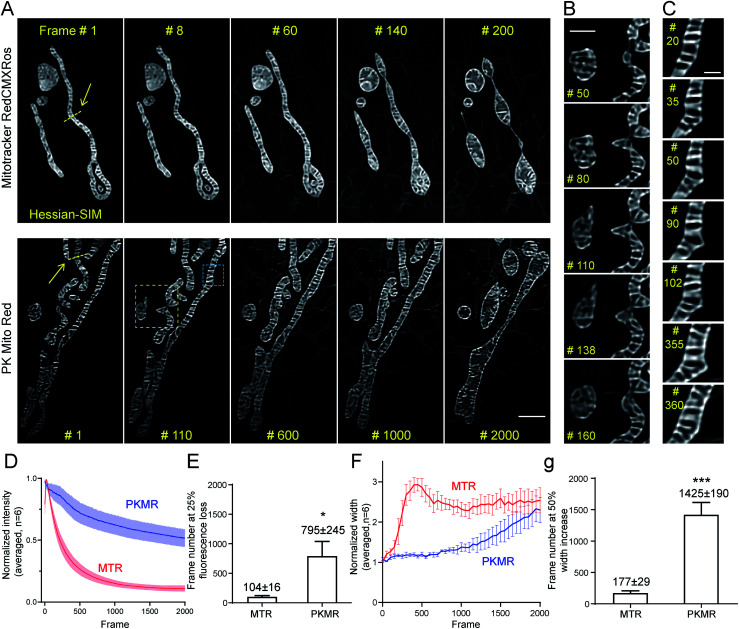
PK Mito Red enables ultra-long time-lapse recording of mitochondrial dynamics. (A) Time-lapse SR imaging of mitochondria in COS-7 cells using Hessian-SIM. Images were acquired 10 fps and processed using the “Enhance Contrast” command in ImageJ to compensate for fluorescence loss over time. (B) Close-up view of the yellow box in (A), highlighting mitochondrial tip-extension–retraction events. (C) Close-up view of the blue box in (A), highlighting dynamic protrusion events. (D) Normalized intensity profiles of time-lapse Hessian-SIM images (*n* = 6). (E) Averaged frame numbers at 25% fluorescence loss. (F) Normalized internal width profile of mitochondria in time-lapse Hessian-SIM images (*n* = 6). (G) Averaged frame numbers at 50% width increase of MTR and PKMR labeled mitochondria. Scale bars: (A) 2 μm, (B) 1 μm, (C) 500 nm.

We then tested whether mitochondrial dynamics could be interrogated for extended durations beyond the diffraction limit using Hessian-SIM, in which the illumination power is minimized compared with other SR microscopy methods. Red dyes are preferred to deep red ones for this application because of their better optical resolution (∼120 nm in 2D mode using 561 nm laser).^6^ Despite the relatively weak illumination power of SIM (7 W cm^−2^, 7 ms exposure time, 100 fps for acquisition, 10 fps for final images; Table S2†), mitochondria in MTR-treated COS-7 cells soon developed a non-specific reticular background at around frame 60 (Fig. 4A and Movie S2†). Severe swelling occurred at frame 140, while the mitochondria rounded up after 200 frames. In contrast, mitochondrial crista structures were clearly visible in COS-7 cells stained with PK Mito Red throughout an observation window of more than 1000 frames, along with minor background fluorescence (Fig. 4A and Movie S2†). To the best of our knowledge, the PK Mito Red – Hessian-SIM approach described here established a record frame number for continuous observation of mitochondrial crista dynamics.

To quantify the photodynamic behavior of the dyes, we analyzed the overall intensity profiles of the images over time. MTR-stained mitochondria rapidly brightened in the first tens of frames, followed by a fast fluorescence loss over the first hundreds of frames (Fig. 4D). The first process was attributed to the release of aggregated cationic dyes due to inner membrane depolarization, a consequence of phototoxicity.^31^ And the subsequent fluorescence loss process reflected photodamage-induced dye diffusion compounded with photobleaching. PKMR-treated mitochondria, in contrast, exhibited a mild and gradual fluorescence decrease throughout the 2000 frames. Frame number at 25% percent fluorescence loss was ∼100 for MTR group and ∼800 for PKMR group (Fig. 4E). Selected line plots across a number of mitochondria were further analyzed. In a typical line plot, the FWHM of Gaussian-fitted peaks were 154 nm and 123 nm, respectively (Fig. S7†), approaching the theoretical resolution of 2D Hessian-SIM. The mitochondrial inner width was measured and plotted for assessing morphological changes over the imaging process. Typical mitochondria stained with PKMR remained their inner width around 500 nm for ∼1200 frames before swelling, while MTR-stained mitochondria started swelling in the first 140 frames (Fig. 4F). On average, PKMR was capable of providing ∼1400 SR images before mitochondria expand to 150% of the original width, while MTR could deliver only ∼170 frames under same conditions (Fig. 4G). Both the morphological and fluorescence intensity data supported PKMR as a steady and reliable marker for maintaining the structural and biochemical integrity of mitochondria under Hessian-SIM.

Thanks to this non-invasive imaging protocol, rich mitochondrial physiological behaviors (as opposed to pathological activitys) were unveiled at unparalleled spatial and temporal resolution. In a typical video of 2000 frames recorded at 10 frames per second, three tip-extension–retraction events were identified (Fig. 4B and Movie S3†). The tip extensions were often less than 1 μm and the dynamic processes lasted 5–10 s. In addition, one kiss-and-run-type mitochondrial interaction was recorded (Movie S2,† Frames 550–750), which lasted for approximately 20 s. Moreover, a mitochondrial hot spot was identified that was particularly active, with dynamic protrusions forming and vanishing several times within 30 s. The duration of these transient events was as short as 1 s (Fig. 4C and Movie S4†). We speculated that these events may be related to the recently discovered mitochondrial nanotunnels^3^ and tubulations^32^ (Fig. S8†), whose identification relied heavily on fixed cells examined with electron microscopy and other SR microscopy methods. The diverse mitochondrial dynamics recorded using PK Mito Red – Hessian-SIM, which occurred over durations of 1 to 20 s and distances less than 2 μm, creates a new paradigm for high-throughput image analysis at high spatial resolution and uncompromised temporal resolution. Such an imaging experiment would not have been possible without reagents with superior optical properties and minimal photodamage.

### PK Mito Red is advantageous in FACS and transplantation of planarian stem cells

Complementing fluorescence imaging, flow cytometry is a fluorescence-based method that provides high-throughput analysis and sorting. Flow cytometry has become a routine assay for the analysis of mitochondrial content, mitochondrial membrane potential dissipation, multidrug resistance, and apoptosis.^28^ In recent years, a number of studies have focused on the relationship between mitochondrial metabolism and stem cell pluripotency, with profound implications for cellular therapy and regenerative medicine.^33–35^ These studies are mainly based on isolation and sorting of sensitive primary cells, setting higher standard for the innocuousness of mitochondrial reagents. An ideal mitochondrial indicator should enable unambiguous identification and harmless sorting of metabolically distinctive stem cell populations for downstream studies.

**Fig. 5:**
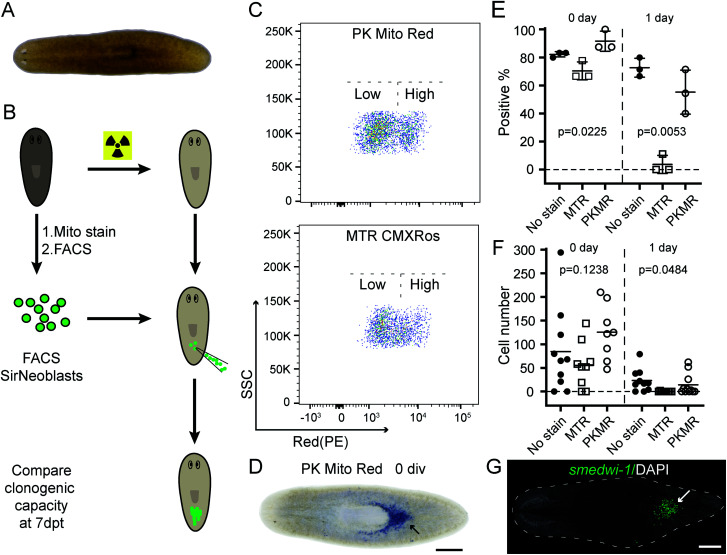
PK Mito Red causes minimal damage during sorting and transplantation of planarian stem cells. (A) Image of a live planarian. (B) Cartoon showing the experimental design of the transplantation assay. (C) FACS plots showing the mitochondria signal intensities of SirNeoblasts after staining with PK Mito Red (upper) and MTR CMXRos (lower), respectively. (D) Representative image showing the clonogenicity of PK Mito Red-stained SirNeoblasts. Transplantation with freshly isolated cells is indicated as 0 div. Blue staining indicates neoblasts generated from transplanted cells (head leftwards). Scale bar: 500 μm. (E) Dot plot of the percentage of planarian hosts 7 days post-transplantation containing *smedwi-1*+ neoblasts generated from cells stained with the indicated dyes. Transplantation with 1 day cultured cells is indicated as 1 day. *N* > 8 in each replicate experiment. (F) Dot plot of the *smedwi-1*+ cell number in each planarian host (*N* > 8). *P* values were calculated using one-way ANOVA. (G) Fluorescence image showing the *smedwi-1*+ neoblasts (green and indicated by an arrow) generated from PK Mito Red-stained SirNeoblasts in a transplant host 7 days post-transplantation. Scale bar: 500 μm.

We therefore tested our mitochondrial probes on planarian stem cells, with a long-term goal of understanding their powerful regenerative capacity that confers planarians with “immortal-like” long lifespans^36^ (Fig. 5A). Despite being an essential organelle for metabolism and aging, the function of mitochondria in planarian stem cell pluripotency and tissue regeneration has not been extensively studied. Adult planarian stem cells, neoblasts, form colonies *in vivo* after being transplanted into stem-cell-depleted planarian hosts (Fig. 5B).^37,38^ By combining SiR-DNA and CellTracker Green indicators to identify the proliferating neoblast population, we have previously established an isolation method for neoblasts, referred to as SirNeoblasts, using FACS.^39^ To leverage the red fluorescence channel, PK Mito Red or MTR were added together with SiR-DNA and CellTracker Green to the isolated planarian cell pellets after maceration, which were then subjected to FACS. PK Mito Red staining clearly distinguished two cell populations of SirNeoblasts that were more distinct than the MTR-stained group (Fig. 5C). Consistently, examination of SirNeoblasts stained with PK Mito Red under a confocal microscope readily identified strongly and weakly fluorescent cells, in contrast to the barely discernible SirNeoblast populations stained with MTR (Fig. S9†). This disparity may be attributed to different mitochondrial membrane potential or to different mitochondrial mass, both of which infer metabolic heterogeneity within SirNeoblasts. The biology behind the different mitochondrial signals in neoblasts is currently under investigation.

Next, we compared the cellular properties of neoblasts isolated using the two mitochondrial dyes. In FACS, cells are first treated with dyes, followed by brief laser irradiations. Both phototoxicity and dark toxicity can possibly affect the viability and stemness of the isolated neoblasts, with the latter one speculated to be dominant. Due to the sensitive nature of stem cells, this assay represents a rigorous test for bio-compatibility of mitochondrial reagents. Compared with unstained SirNeoblasts, PK Mito Red staining did not reduce the viability or nature of stem cells *in vitro*, as indicated by the pan stem cell marker *smedwi-1* (Fig. S10B†). MTR staining, however, slightly but significantly reduced the viability but not the nature of stem cells (Fig. S10A†). Freshly isolated PK Mito Red-stained cells (0 div) had a colony-formation rate of 92% in planarian hosts similar to that of unstained SirNeoblast transplantation,^39^ but better than the ∼70% success rate of MTR-stained cells (Fig. 5D and E). When the isolated SirNeoblasts were cultured for 1 day before transplantation, a colony-formation rate of ∼55% was retained for PK Mito Red-stained cells compared to 4% for MTR-stained cells (Fig. 5E). The cell numbers were then counted using fluorescent whole-mount *in situ* hybridization (Fig. 5G). The colony sizes were similar among all three groups but exhibited a slightly lower number in planarians transplanted with 0 div SirNeoblasts labeled with MTR. Later, when 1 day SirNeoblasts were used for transplantation, the planarians that received MTR-stained cells performed the worst, while those receiving PK Mito Red-stained cells performed comparably to those receiving unstained SirNeoblasts (Fig. 5F). Altogether, compared to MTR, PK Mito Red offers a minimally disruptive assay for sorting SirNeoblasts with different mitochondrial states while maintaining maximal stemness. This effect is particularly significant in the neoblasts after 1 day of *in vitro* culture, highlighting the marked innocuousness of PK Mito Red in stem cell research.

## Discussion

The main focus of this work was to tackle the phototoxicity of imaging probes, a commonly overlooked feature in bioimaging.^25^ Synthetic fluorophores are favored over genetically encoded fluorescent proteins in a number of applications thanks to their superior brightness and convenient use. In super-resolution imaging where fluorophore brightness is of pivotal importance and in primary-cell analysis where genetic manipulations are cumbersome (if not impossible), organic dyes are indispensable reagents. In recent years, innovation in bioimaging dyes has mainly focused on brightness,^27,40,41^ fluorogenicity,^42,43^ photostability,^7,44,45^ and photo-switching.^5^ Despite similarities in their mechanism, the distinctions between phototoxicity and photobleaching are important in fluorophore design.^46^ Although they both stem from the excited states of chromophores, photobleaching primarily concerns the destruction of the chromophore, while phototoxicity is believed to relate to the sensitization of ROS and subsequent reactions with biomacromolecules (Fig. 1B). A fluorophore could be engineered to be stable enough to tolerate the ROS species generated by itself, but such modification may not stop ROS species from disrupting biomacromolecules. Therefore, combating phototoxicity, to a certain extent, is more challenging than enhancing photostability. Common strategies for enhancing photostability, such as electron-withdrawing group introduction, have not been proven effective to alleviate photodamage. Meanwhile, due to the electrochemical-potential-driven localization of mitochondrial dyes, fluorescence loss in live-cell mitochondrial imaging is compounded by both photobleaching and membrane-potential loss, a symptom of photodynamic damage.^31^ Therefore, special care should be taken in analysing mitochondrial imaging data regarding photobleaching and phototoxicity. As such, the comparison between Cy3-bisNB (**3**) and Cy3-bisCOT (**4**) is particularly relevant. **3** is more photostable than **4**; however, the phototoxicity of **3** appeared to be similar to its parental compound, **1**, and was significantly higher than **4** (Fig. 1E and F). This clearly suggests that reducing phototoxicity and suppressing photobleaching should be evaluated independently.

In the context of photochemistry and molecular design, this work represents a major development in self-healing dyes for mitochondrial imaging in live cells. Covalent conjugation of triplet-state quenchers to fluorophores has been proposed to exhibit reduced phototoxicity according to *in vitro* ROS measurements.^20^ This work, along with previous studies, provides firm evidence that self-healing strategy could effectively alleviate phototoxicity when used for live cell imaging applications. An interesting finding in this work suggested that COT effectively reduces phototoxicity, while nitrobenzene, another popular TSQ in reducing photobleaching, does not alleviate photodamage. This result is in accordance with the triplet state lifetime and singlet oxygen generation of Cy5-COT measured by Blanchard *et al.*^20,30^ We tentatively attribute this discrepancy to their different mechanisms of triplet-state quenching,^30,47,48^ in which COT quenches triplet-state through an energy transfer process, while nitrobenzene works through a redox process. A future extension of this strategy would be the upgrade of the dyes to immobilizable versions for permanent labelling of mitochondria.^49^ Additionally, the findings in this work could potentially inspire the development of advanced probes for various organelles and protein/nucleic acid targets, as well as next generation TSQs. A recent perspective conveys several opinions on self-healing dyes as a strategy for reducing photobleaching.^16^ We have demonstrated here that reducing phototoxicity represents a new direction for self-healing dyes.

Considering instrumentation, PK Mito dyes have been tested on a range of fluorescence microscopes, including epifluorescence microscope, high-content imager, spinning-disk confocal microscope, and Hessian-SIM. We recommend Hessian-SIM as a synergistic partner of PK Mito dyes due to its strictly budgeted photon flux compared with other SR imaging methods.^6^ Hessian-SIM can achieve a spatial resolution of ∼120 nm (2D mode using 561 nm excitation) and a temporal resolution of up to 188 Hz. With PK Mito Red, the effective observation window could reach more than 1000 frames. In comparison to other SR imaging methods, our combination is advantageous in not only biocompatibility, but unveils richer mitochondrial inner membrane and crista dynamics spanning different time scales. For example, the observed kiss-and-run fusion lasted 20 s, while a transient dynamic protrusion lasted for only 1 s. These events are reported for the first time in live cells at super-resolution level. Compared to Mito PB Yellow dye – STED imaging methods that offered ∼60 nm spatial and ∼1 s temporal resolutions,^7^ our approach is advantageous in capturing both fast dynamics and long-duration events, therefore significantly expanding the observation windows of biological processes. In principle, however, PK Mito dyes should generally reduce the photodamage during image acquisitions under a variety of fluorescence microscopies. We expect that the PK Mito dyes could also be applied to STED microscopy,^50^ multi-photon microscopy,^51^ and light sheet microscopy.^52^ Compatibility with single-molecule localization-based SR imaging^53^ may also be realized after integrating photoactivation/photoswitching modules to the dyes. Finally, we would like to highlight the sorting and transplantation experiments on planarian cells as a closed-loop demonstration of the general toxicity of a new dye.^22^ In our work, the upgrading of probe molecules and development of instrumentations evolve in parallel, representing a new trend in modern biotechnology.^24,54^ With the emergence of these biocompatible fluorescent probes, we look forward to their impact on novel cytometry instruments.

In summary, we present COT-conjugated carbocyanine dyes as mitochondrial probes with minimal photodamage and toxicity. They maximally retain the health of mitochondria in live-cell volumetric imaging of cardiomyocytes, in remarkably long time-lapse SR imaging with Hessian-SIM, and in planarian stem-cell sorting for regeneration. We envision a role for these probes in revealing the dynamics of mitochondrial morphology and interactions, as well as mitochondria-related biotechnologies. Methodologically, as a pilot study on mitochondria, it also paves the way for using triplet-state depleted dyes to upgrade the toolkit for non-disruptive investigation of various cellular targets.

## Materials and methods

For chemical synthesis of the probes, spectral measurements, photobleaching experiment, cell culture, mitochondrial staining, cardiomyocyte isolation, phototoxicity assay, and 3D imaging, Hessian-SIM setup, planarian cell sorting and transplantation experiments, see ESI.†

### HeLa cell phototoxicity assays

HeLa cells were incubated with 250 nM selected dye (or without dye in light only group) in 1 mL DMEM (Macgene, CM10017) for 15 min at 37 °C with 5% CO_2_. Then, the cells were washed three times with fresh media, and kept in DMEM for phototoxicity test. Treated cells were placed on an epifluorescence microscope (EVOS FL imaging system, ThermoFisher Scientific) and were illuminated with a 40× objective (ThermoFisher AMEP4699, 0.75 NA, EVOS) at specific intensities for different time periods. Light sources: GFP channel-EVOS GFP LED light cube (AMEP4651, Ex: 470/22 nm, Em: 510/42 nm, 2.5 W cm^−2^), RFP channel-EVOS RFP LED light cube (AMEP4652, Ex: 531/40 nm, Em: 593/40 nm, 1.7 W cm^−2^), Cy5 channel-EVOS Cy5 LED light cube (AMEP4656, Ex: 628/40 nm, Em: 693/40 nm, 1.9 W cm^−2^). Intensities of light source after 40× objective were measured using a SANWA LP1 laser power meter. After illumination, the samples were maintained in an incubator for 2 h and then treated with 10 μM propidium iodide (PI) in DMEM for 5 min. Then the cells were imaged on the same microscope using a 10× objective (ThermoFisher AMEP4623, 0.30 NA, EVOS) to assess cell phototoxicity. Cells exhibiting nuclear fluorescence under RFP channel were scored as dead and the total cell number in the illumination area was counted under bright field. The fraction of live cells was calculated as cell viability.

### Hessian-SIM imaging

COS-7 cells were incubated on pre-treated coverslips (see ESI†) with 250 nM MitoTracker Green FM (Thermo Fisher Scientific, M7514), MitoTracker Red CMXRos (Thermo Fisher Scientific, M7512) or PK Mito Red in HBSS containing Ca^2+^ and Mg^2+^ but no phenol red (Thermo Fisher Scientific, 14025076) at 37 °C for 15 min, then the cells were washed 3 time with fresh medium, and kept in fresh medium for imaging. Hessian-SIM set up in ref. 6 was applied for SIM imaging. Detailed imaging parameters are listed in Table S2.† The images were processed using MATLAB and ImageJ.

## Author contributions

Z. C. and L. C. conceived the project. Z. C. and Z. Y. designed PK Mito dyes, and Z. Y. performed the chemical synthesis. Z. Y. measured the spectra and performed the phototoxicity assay on HeLa cells. L. L. designed and performed the confocal imaging of cardiomyocytes and Hessian-SIM imaging experiments and analyzed the images. L. J. performed the cardiomyocyte phototoxicity experiments. T. L. performed the co-localization, CCCP, and MTT toxicity assays. X. H. assisted with the Hessian-SIM experiments. Yan Z. isolated cardiomyocytes. K. L. designed and performed the planarian experiments with Y. Y. and Yun Z. L. C. supervised the confocal and SIM experiments. K. L., L. C., and Z. C. wrote the manuscript with input from all authors.

## Conflicts of interest

Z. C., Z. Y., and L. L. have submitted a patent application based on the mitochondria dyes described in this work.

## Supplementary Material

SC-011-D0SC02837A-s001

SC-011-D0SC02837A-s002

SC-011-D0SC02837A-s003

SC-011-D0SC02837A-s004

SC-011-D0SC02837A-s005
