# Supplementary material for: Cyclooctatetraene-conjugated cyanine mitochondrial probes minimize phototoxicity in fluorescence and nanoscopic imaging
Source: Chem Sci. 2020 Jul 27;11(32):8506–16. doi: 10.1039/d0sc02837a (PMC8161535; doi:10.1039/d0sc02837a)
Supplement: SC-011-D0SC02837A-s001 [file SC-011-D0SC02837A-s001.pdf]

Electronic Supplementary Material (ESI) for Chemical Science.
This journal is © The Royal Society of Chemistry 2020

Supplementary Information for

Cyclooctatetraene-conjugated cyanine mitochondrial probes minimize phototoxicity in fluorescence and nanoscopic imaging

Zhongtian Yang[a,b,†], Liuju Li[a,c,†], Jing Ling[a,b], Tianyan Liu[a,b], Xiaoshuai Huang[a,b], Yuqing Ying[d,e], Yun Zhao[d,e], Yan Zhao[a,b], Kai Lei*[d,e], Liangyi Chen*[a,c,f] and Zhixing Chen*[a,b,f]

a. *Institute of Molecular Medicine, Beijing Key Laboratory of Cardiometabolic Molecular Medicine, Peking University, Beijing, China.*

b. Peking-Tsinghua Center for Life Sciences, Peking University, Beijing, China.

c. State Key Laboratory of Membrane Biology, Peking University, Beijing, China

d. Zhejiang Provincial Laboratory of Life Sciences and Biomedicine, Key Laboratory of Growth Regulation and Transformation Research of Zhejiang Province, School of Life Sciences, Westlake University, Hangzhou, Zhejiang, China

e. Institute of Biology, Westlake Institute for Advanced Study, Hangzhou, Zhejiang Province, China

f. PKU-Nanjing Institute of Translational Medicine, Nanjing, China

† *These authors contributed equally to this work*

**To whom correspondence should be addressed. E-mail: zhixingchen@pku.edu.cn (Z.C.); lychen@pku.edu.cn (L.C); leikai@westlake.edu.cn (K.L.)*

Index

1.Tables S1 to S2 .... S2
2.Figures S1 to S10 .... S4
3.Captions for movies S1 to S4 .... S11
4.Supplementary text S12
5. Chemical synthesis S18
6. References of supplementary materials S32

Other supplementary materials for this manuscript include the following:

Movies S1 to S4

Supplementary tables and figures

Table S1. Photophysical Data of mitochondrial dyes

Compound	Solvent	λ_{abs}/nm	λ_{em}/nm[a]	Φ_f[b]
**PK Mito Red**	MeOH	549	569	0.12
	DMSO	556	580	0.16
	CH_3CN	550	567	0.25
	Toluene	562	580	0.50
	PBS	548	564	0.08
**PK Mito Deep Red**	MeOH	644	670	0.12
	DMSO	650	680	0.10
	CH_3CN	643	668	0.08
	Toluene	652	695	0.05
	PBS	653	661	0.02

$\varepsilon_{\text{PK Mito Red, MeOH}} = 1.7 \times 10^5\ M^{-1}\cdot cm^{-1}$, $\varepsilon_{\text{PK Mito Deep Red, MeOH}} = 2.6 \times 10^5\ M^{-1}\cdot cm^{-1}$.

Maximum emission wavelengths were measured with 510 nm extinction light for PK Mito Red and 610 nm light for PK Mito Deep Red.

Quantum yields were measured with Rhodamine B in DMSO as reference (Φ=0.65), The fluorescence quantum yield, Φ_f (sample), were calculated according to equation as following:

$$\frac{\Phi_{f,sample}}{\Phi_{f,ref}} = \frac{OD_{ref} \cdot I_{sample} \cdot d^2_{sample}}{OD_{sample} \cdot I_{ref} \cdot d^2_{ref}}$$

Φ_f: quantum yield of fluorescence;

I: integrated emission intensity;

OD: optical density at the excitation wavelength;

d: refractive index of solvents, d_{DMSO}=1.478; $d_{methanol}$=1.329; d_{water}=1.33; $d_{toluene}$=1.497; $d_{acetonitrile}$=1.343.

Table S2. Dyes and parameters used in imaging and phototoxicity experiments.

	Label	Wave-length (nm)	Light intensity (mW)	Expo-sure time	Illumination intensity (W/cm^2)
**Fig. 1*A***	MitoTracker Green FM	488	0.12	5 ms	14
**Fig. 1*E***	Compound **1-4**	531	8.7	1-30 min	1.7
**Fig. 2*C***	MitoTracker Red CMXRos	531	8.7	1-5 min	1.7
	PK Mito Red	531	8.7	1-30 min	1.7
**Fig. 2*D***	MitoTracker Deep Red FM	628	9.5	1-5 min	1.9
	PK Mito Deep Red	628	9.5	1-30 min	1.9
**Fig. 3*A***	MitoTracker Red CMXRos	568	5.98	5 s × 68	1.4
	PK Mito Red	568	5.98	5 s × 68	1.4
**Fig. 3*A***	MitoTracker Deep Red FM	633	5.05	5 s × 69	1.2
	PK Mito Deep Red	633	5.05	5 s × 69	1.2
**Fig. 3*B*, *C*, *D* Movie S1**	Hoechst	405	1.6×10^{-2}	200 ms	0.12
	ViaFluor® 488 Live Cell	488	7.7×10^{-3}	200 ms	0.06
	Microtubule Stain LysoView 540	561	8.7×10^{-2}	200 ms	0.66
	PK Mito Deep Red	640	1.7×10^{-2}	200 ms	0.13
**Fig. 4*A*, *B*, *C* Movie S2, 3, 4**	MitoTracker Red CMXRos	561	0.06	7 ms	7
	PK Mito Red	561	0.06	7 ms	7
**Fig. S8**	PK Mito Red	561	0.06	7 ms	7

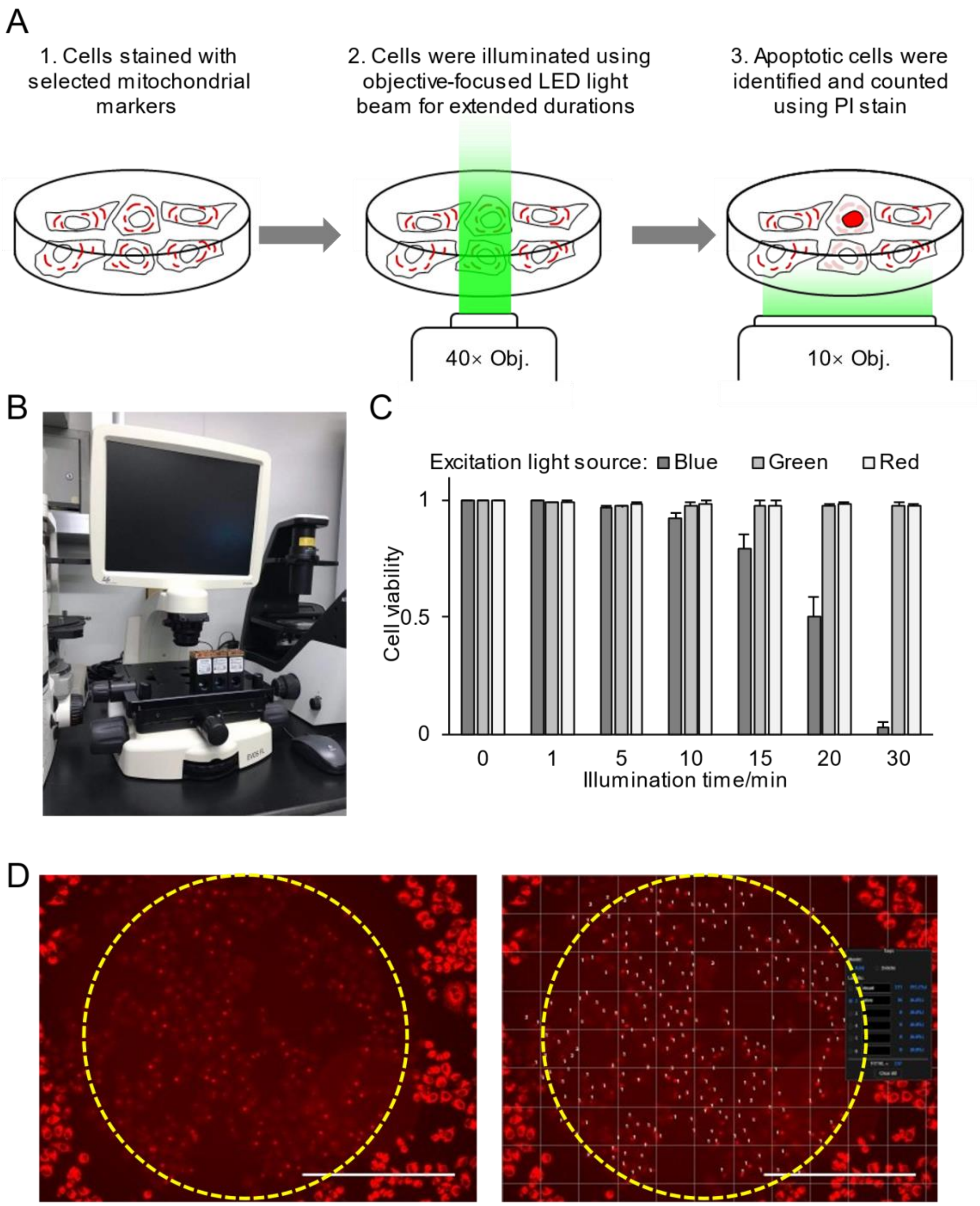

Fig. S1. Phototoxicity measurement of HeLa cells. (A) Schematic illustration of the procedure to measure phototoxicity of mitochondrial dyes using PI as cell death marker. (B) The EVOS FL imaging system and LED light cubes used for phototoxicity experiment. (C) Comparison of excitation light induced cytotoxicity (2.5 W/cm^2 for blue light, 1.7 W/cm^2 for green light, 1.9 W/cm^2 for red light). (D) Representative image and cell counting. The circle marked the illuminated area of the 40× objective, note the reduced brightness of mitochondria and the emerged PI signal of the nucleus in the circle. Scale bars, 400 μm.

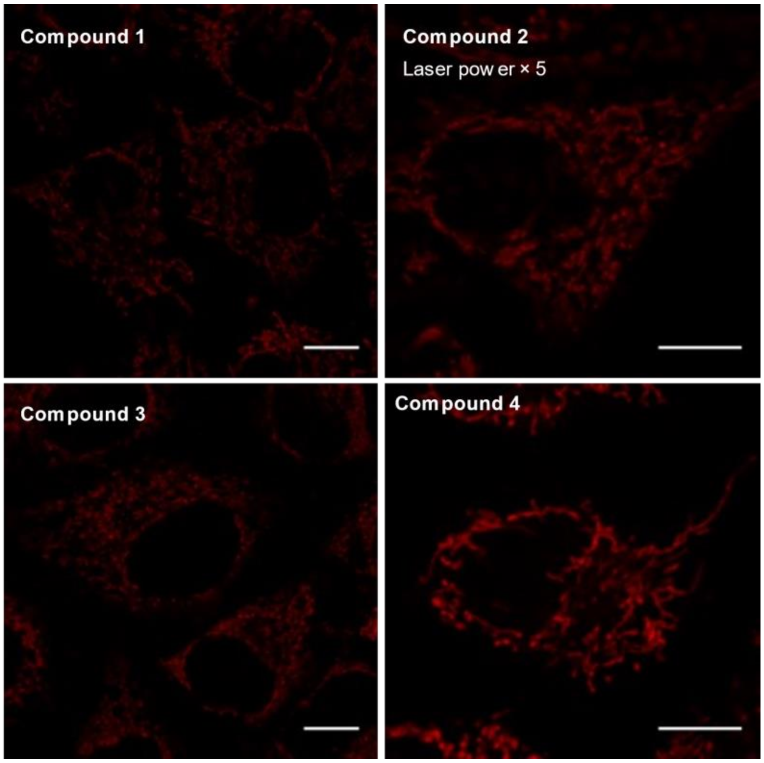

Fig. S2. The confocal images of compound **1-4** labeled HeLa cells. Laser power used for Compound **2** was 5 times stronger than others. Scale bars, 10 μm.

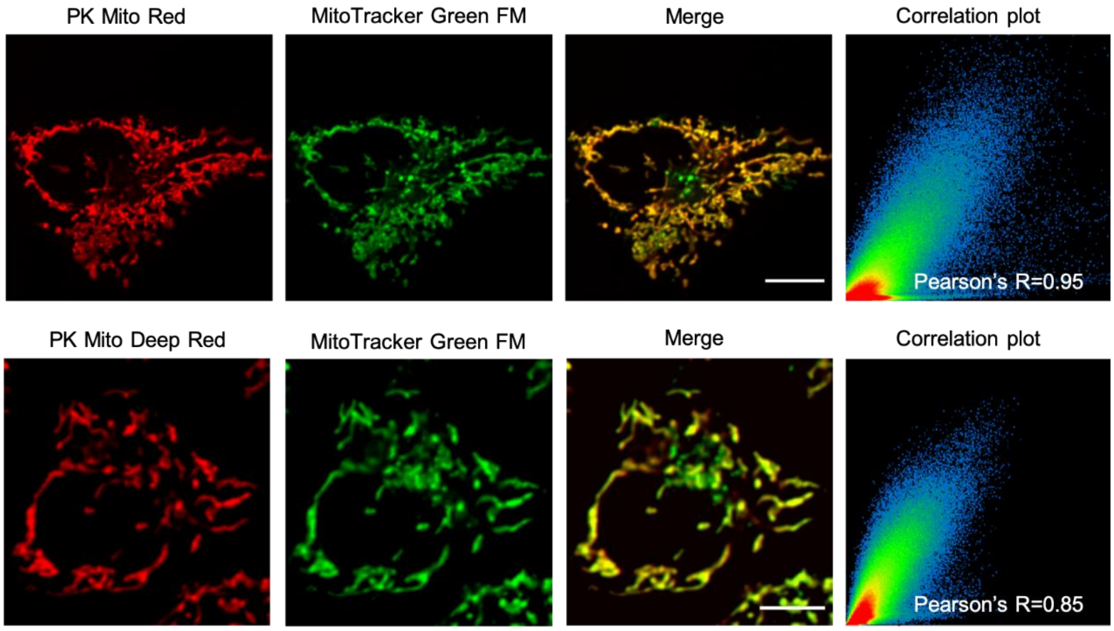

Fig. S3. Co-localization analysis of live HeLa cells stained with PK Mito Red, PK Mito Deep Red and MitoTracker Green FM. From left to right: PK Mito Red (λ_{ex} = 550 nm; λ_{em} = 570 ± 20 nm) or PK Mito Deep Red (λ_{ex} = 640 nm; λ_{em} = 670 ± 20 nm); MitoTracker Green FM (λ_{ex} = 488nm; λ_{em} = 516 ± 20 nm); merged image; Pearson correlation coefficient plot of two channels. Scale bars, 10 μm.

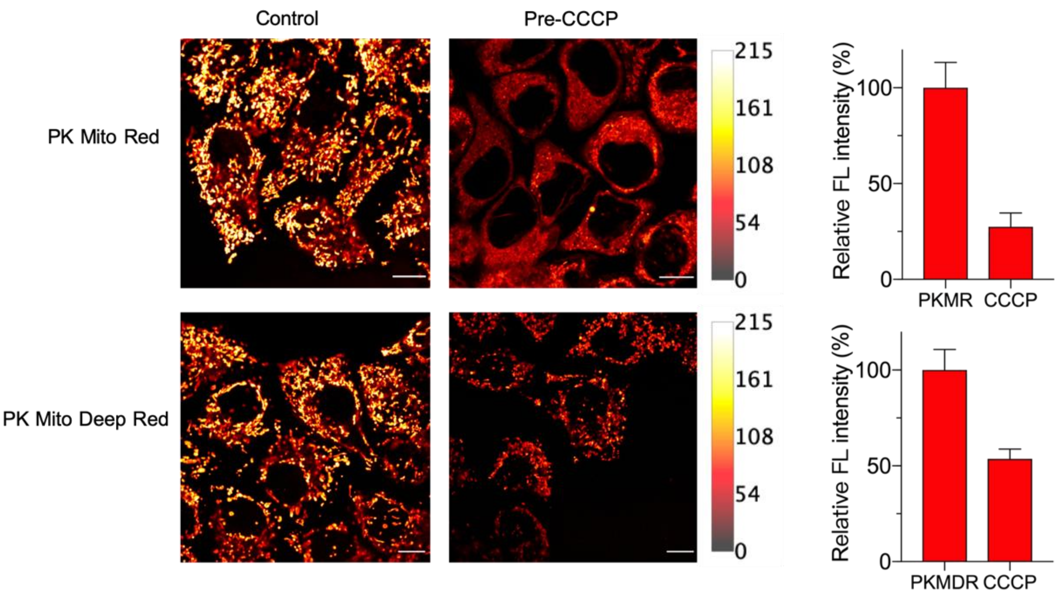

Fig. S4. The confocal images of live HeLa cells pretreated with CCCP prior to staining with PK Mito dyes: control experiment (left), CCCP pretreatment (middle), and their relative fluorescence intensity (right, n = 20). Scale bars, 10 μm.

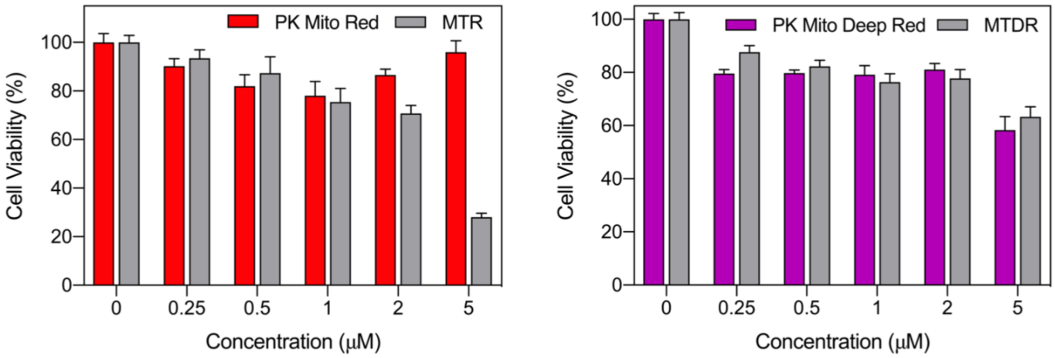

Fig. S5. Cell viability measurements of the HeLa cells stained with PK Mito Red and PK Mito Deep Red by MTT assay. The results are expressed as percentages of the dye-free controls. All data are presented as mean ± S.D. (n = 5).

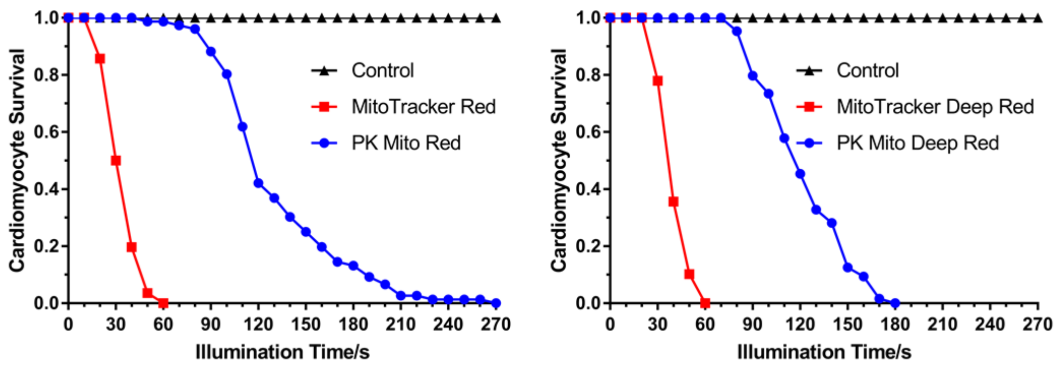

Fig. S6. Survival rates of the cardiomyocytes irritated with laser (568-nm or 633-nm). The cardiomyocytes were pre-treated (or untreated in control group) with 250 nM PK Mito dyes or MitoTracker dyes.

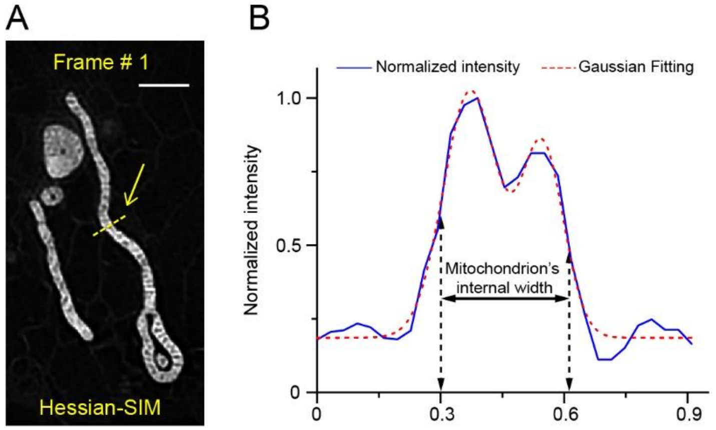

Fig. S7. (A) A Hessian-SIM image of mitochondria in COS-7 cells. (B) Fluorescence intensity profile of MitoTracker Red CMXRos along the line in (A), which can be fitted by bi-Gaussian function and the distance between the half value of the peak of bi-Gaussian curve indicates the mitochondrial width.

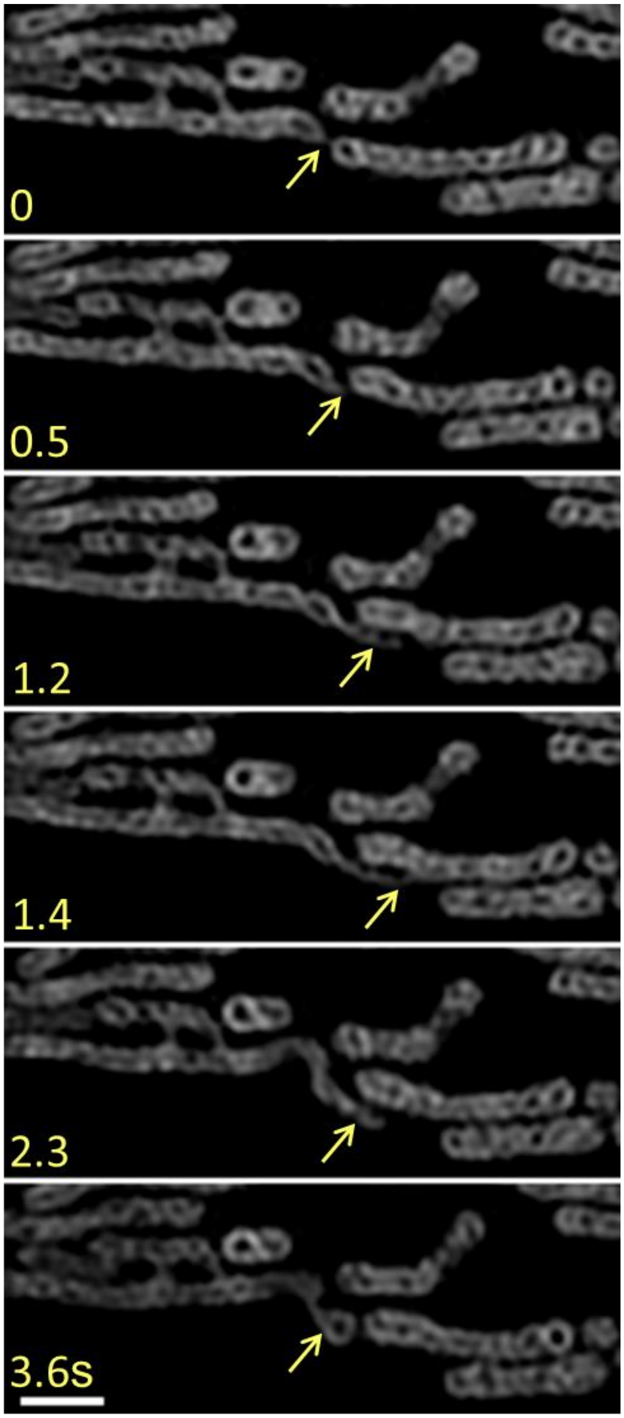

Fig. S8. A dynamic tubulation event of mitochondria. A COS-7 cell with mitochondrial inner membrane labeled by PK Mito Red was imaged under Hessian-SIM. Yellow arrows indicate the formation and retraction of dynamic tubules. Scale bar, 1 μm.

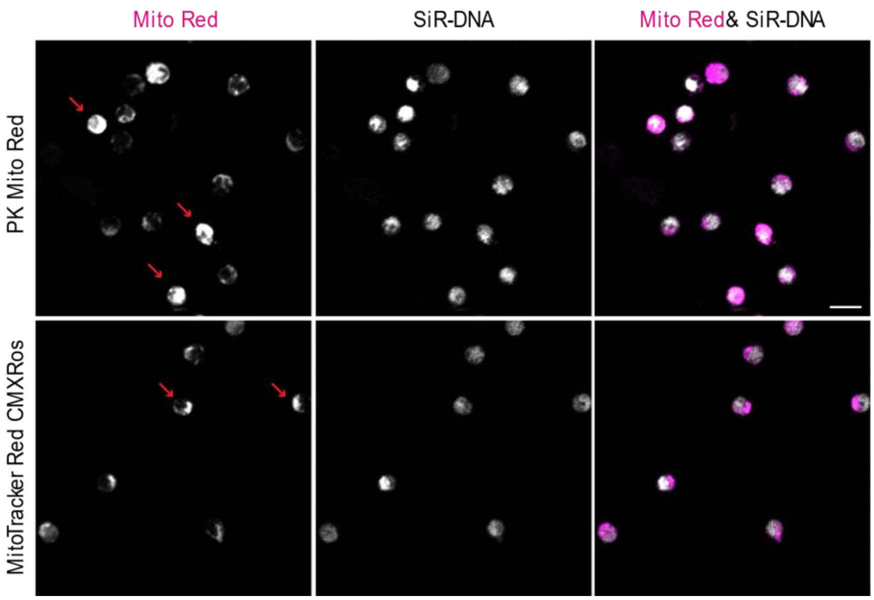

Fig. S9. Epifluorescence images showing the mitochondria staining of SirNeoblasts using PK Mito Red (upper) and MTR CMXRos (lower), respectively. Red arrows indicate cells with stronger mitochondrial signals. Note the cells labeled with PKMR showed two distinctive populations, while the MTR labeled ones showed a relatively continuous distribution of mitochondrial signal. Scale bar, 20 μm.

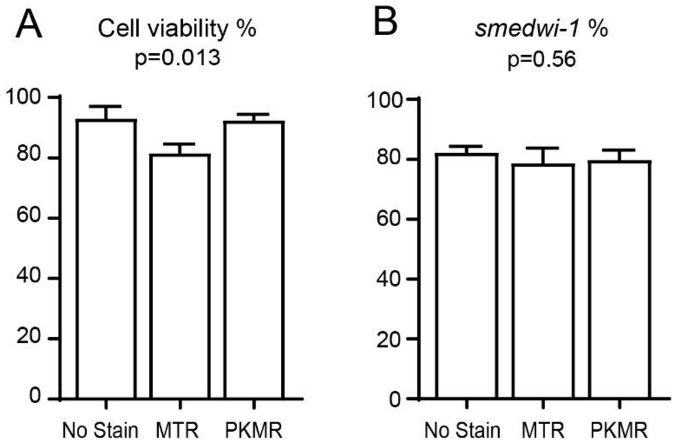

Fig. S10. Viability and nature of stem cells of PK Mito Red or MTR CMXRos stained SirNeoblasts. (a) Bar plot presenting the viability of SirNeoblasts unstained or stained with PK Mito Red and MTR CMXRos, respectively, after 1-day culture in KnockOut DMEM containing 5% FBS. (b) Bar plot presenting the percentage of *smedwi*-1+ SirNeoblasts unstained or stained with PK Mito Red and MTR CMXRos, respectively, after 1-day culture in KnockOut DMEM containing 5% FBS. P value was calculated using one-way ANOVA test.

Captions for Movies S1 to S4

Movie S1

Three-color 3D-rendered volumetric views of cardiomyocyte imaged with spinning disk confocal microscopy. Adult rat cardiomyocytes were labeled with MitoTracker Deep Red FM/PK Mito Deep Red (red), Hoechst (blue) and LysoView 540 (green).

Movie S2

Comparison between MitoTracker Red CMXRos and PK Mito Red under Hessian-SIM imaging of mitochondrial dynamics in COS-7 cells. Scale bar: 2 μm.

Movie S3

Mitochondrial tip extension-retraction events. Close-up view of mitochondria tipping events indicated by asterisk characters. Scale bar: 500 nm (2 μm in the first frame).

Movie S4

Mitochondrial inner membrane dynamics. Close-up view of an active spot of mitochondrial inner membrane highlighting transient protrusion events. Scale bar: 200 nm (2 μm in the first frame).

Supplementary Text

Supplementary methods

UV-vis and fluorescence spectroscopy. UV-Vis absorption spectra of sample solutions in spectral grade solvents were measured using a Shimadzu UV-1780 UV-Vis spectrophotometer in a 1 cm square quartz cuvette. Emission spectra were measured using a Shimadzu RF-5301PC spectrofluorometer. Extinction coefficients of mitochondrial dyes in methanol were measured using a Shimadzu UV-1780 UV-Vis spectrophotometer in a 1 cm square quartz cuvette. The absolute quantity of dyes was quantified using ^{1}H NMR with 2.0 μL toluene as internal reference.

Cell maintenance and preparation. Human cervical carcinoma cell line HeLa cells were cultured in high-glucose DMEM (Macgene, CM10017) medium containing 10% heat-inactivated fetal bovine serum (VISTECH, SE100-011) and 1% penicillin sulfate and streptomycin (Macgene, CC004). The cells were cultured in an incubator at 37 ℃ with 5% CO_2. COS-7 cells were cultured in high-glucose DMEM (GIBCO, 21063029) supplemented with 10% fetal bovine serum (FBS) (GIBCO) and 1% 100 mM sodium pyruvate solution (Sigma-Aldrich, S8636) in an incubator at 37 ℃ with 5% CO_2 until ~75% confluency was reached. Human foreskin fibroblast cells were cultured in high-glucose DMEM (GIBCO, 21063029) supplemented with 20% FBS (GIBCO) in an incubator at 37 ℃ with 5% CO_2 until ~75% confluency was reached. For dark toxicity experiments, cells were seeded into a flat-bottomed 96-well plate (Corning, 3599). For phototoxicity experiments, cells were seeded in dishes (Corning, 430165). For confocal imaging, cells were seeded in glass bottom dishes (Nest, 801001). For Hessian-SIM imaging experiments, cells were seeded onto coverslips (Thorlabs, CG15XH).

Photobleaching assays. Samples were prepared by spin coating (~4000 rpm, SW-4A spin coater, Setcas) of a 400μL dye solution (5μM, Cy3, **1**, **2**, PK Mito Red, MTR) in Milli-Q water, each containing 5% PVA (Yuanye, S30196, 1750 ± 50) on cleaned coverslips [1].The samples were then irradiated using Zeiss LSM 710 microscope with a 20 × objective (Plan-Apochromat 20x/0.8 M27). Three groups of time-lapse images (100 frames, 1fps, 100% laser power) were acquired for each dye, and mean intensity of ROIs were calculated using ZEN. Normalized intensities of images with error bars were plotted as photobleaching curves.

Confocal imaging of compound 1-4 labeled HeLa cells. HeLa cells were seeded in glass bottom dishes and subjected to subsequent experiments at a cell density of 70-80%. HeLa cells were stained

250 nM Compound **1-4** for 15 min. The cells were then washed with PBS (3 × 1 mL per dish) and 1 mL of medium was added to each dish for imaging assay. Confocal images were acquired using Zeiss LSM 710 with a 63 × oil-immersion objective (Plan-Apochromat 63× /1.40 Oil DIC M27). The incubated cells were excited at 550 nm, and the emission signals were collected at 570 ± 20 nm.

Dark toxicity assays. The effect of PK Mito dyes and commercial mitochondrial dyes on cell viability (without light irradiation) was analyzed using 3-(4,5- dimethylthiazol-2-yl)-2,5-diphenyltetrazolium bromide (MTT, SIGMA, M2128-1G). HeLa cells were seeded into a flat-bottomed 96-well plate (1×10^4cells/well) and incubated in cell culture medium (DMEM containing 10% FBS and 1% penicillin-streptomycin liquid) at 37 ℃ in a 5% CO_2 incubator for 24 h. The medium was then replaced with a culture medium containing various concentrations (0, 0.25, 0.5, 1.0, 2.0 and 5.0 μM) of PK Mito dyes and MitoTracker Red CMXRos (MTR, Invitrogen, M7512), MitoTracker Deep Red FM (MTDR, Invitrogen, M22426). After staining for 12 h at 37 ℃, the medium was replaced with a fresh medium. MTT reagent (final concentration, 0.5 mg/mL) was added to each well, and the plates were incubated for another 4 h in a CO_2 incubator. The supernatant was then removed and 100 μL of DMSO was added to dissolve the formazan crystals. And the cell culture plate was shaken for 10 min until no particulate matter was visible. Absorbance in each well was measured at 492 nm using a microplate reader (TECAN Infinite M Nano+, Switzerland). The cell viability (%) was calculated according to the following equation: cell viability % = A/B ×100, where A represents the optical density of the wells treated with various concentration of the PK Mito dyes and B represents that of the wells treated with medium.

Colocalization assays. HeLa cells were seeded in glass bottom dishes and subjected to subsequent experiments at a cell density of 70-80%. Hela cells were stained 250 nM PK Mito dyes (PK Mito Red, PK Mito Deep Red) for 15 min and 100 nM commercial mitochondria dye (MitoTracker Green FM, Invitrogen, M7514) for 15 min. The cells were then washed with PBS (3 × 1 mL per dish) and 1 mL of medium was added to each dish for imaging assay. Confocal images were acquired using Zeiss LSM 710 with a 63 × oil-immersion objective (Plan-Apochromat 63× /1.40 Oil DIC M27). The incubated cells were excited at 488 nm for MitoTracker Green FM, 550 nm for PK Mito Red, and 640 nm for PK Mito Deep Red with semiconductor lasers, and the emission signals were collected at 516 ± 20 nm for MitoTracker Green FM, 570 ± 20 nm for PK Mito Red, and 670 ± 20 nm for PK Mito Deep Red, respectively.

Cellular uptake mechanism assay. Carbonyl cyanide m-chlorophenylhydrazone (CCCP, Targetmol, T7081) can collapsed mitochondrial membrane potential. CCCP test was carried out to confirm that whether the mitochondria-targeting properties was dependent of the membrane potential [2]. For control group, Hela cells were stained 250 nM PK Mito dyes for 15 min; for CCCP treatment group, Hela cells were pre-cultured 20 μM CCCP for 20 min then labeled by PK Mito dyes in the same way. Confocal photography was acquired by Leica TCS SP8 confocal microscopy and 100×oil-immersion objective (HC PL APO 100×/1.40 Oil CS2) and ROI intensity analysis was conducted by ImageJ.

Image processing and analysis for colocalization and cellular uptake assays. Micrographs were processed and analyzed by ImageJ 1.48 v (32-bit). Quantification of the fluorescence intensity was achieved via Analyze >> Tools >> ROI manager in ImageJ from three parallel experiments. Quantification of colocalization ecoefficiency was achieve via an external plugin via Plugins >> Colocalization Finder. For more details, please refer to online sources: https://imagej.nih.gov/ij/

Isolation and culture of adult rat cardiomyocytes. All procedures conformed to the Guide for the Care and Use of Laboratory Animals published by the US National Institutes of Health (NIH Publication No. 85-23, revised 1996) and the rules of the American Association for the Accreditation of Laboratory Animal Care International and were approved by the Institutional Animal Care and Use Committee of Peking University (accredited by Association for Assessment and Accreditation of Laboratory Animal Care international). Adult male Sprague–Dawley rats weighing 150-180 g were anesthetized by intraperitoneal injection of 10% trichloroacetaldehyde monohydrate (0.3 mL/mg). Single ventricular myocytes were enzymatically isolated from the hearts. Freshly isolated cardiomyocytes were plated on laminin-coated (Sigma, 11243217001) culture dishes (or 24-well culture plates for phototoxicity experiments) for 1 h and the attached cells were then maintained in M199 media (Sigma, M3769-1L) as described previously [3].

Phototoxicity assay of cardiomyocytes. Freshly isolated cardiomyocytes were plated on 24-well glass bottom plates (Cellvis, P24-1.5H-N). To stain the cardiomyocytes, media were replaced with fresh media containing 250 nM Mito dyes (MitoTracker Red CMXRos, PK Mito Red, MitoTracker Deep Red FM, PK Mito Deep Red). After incubating for 15 min at 37 ℃, the cells were then washed with PBS three times before maintained in fresh media for subsequent imaging analysis. Cardiomyocytes pre-treated with Mito dyes were analyzed using an Opera Phenix high-content imaging system (PerkinElmer) with a 20 ×/0.4 NA air objective. A 568-nm laser (5.98 mW after

objective) paired with a 570-620 nm bandpass emission filter, and a 633-nm laser (5.05 mW after objective) paired with a 655-705 nm bandpass emission filter, were used to irradiate and image red and far-red dye-stained cells, respectively. Imaging and irradiation were performed under wide-field mode. Area of illuminated region was 0.42 mm^2. The average light intensities on cells were calculated to be 1.4 W/cm^2 (568 nm) and 1.2 W/cm^2 (633 nm). In each imaging cycle, cells were irradiated continuously with laser for 5 s, followed by collection of a fluorescence image (exposure time $\leqslant$10 ms) and a bright field image (exposure time $\leqslant$10 ms). Cardiomyocytes were defined as surviving before irreversible contraction started. The survival time of cardiomyocytes were manually counted based on bright field images (Figure S6).

Confocal imaging of cardiomyocytes. For confocal imaging experiments, adult rat cardiac myocytes were seeded onto coverslips (Thorlabs, CG15XH) and stained with Hoechst (Thermo Fisher Scientific, H1399), 1× ViaFluor 488 Live Cell Microtubule Stain (Biotium, 70062-T), 1× LysoView 540 (Biotium, 70061) and 250 nM PK Mito Deep Red/MTDR for 15 min before washed with fresh media. Confocal imaging was acquired on an Olympus IX81 microscope with a 100×, 1.35 NA oil immersion objective, a scanning confocal system (Yokogawa, CSU-X1) and four laser beams of 405 nm, 488 nm, 561 nm and 647 nm. The images were captured by a sCMOS (Flash 4.0 V3, Hamamatsu, Japan). Detailed imaging parameters are listed in Table S2.

Preparation of coverslip for Hessian-SIM. To clean the coverslips for live-cell imaging, we immersed the coverslips in 10% Powdered Precision Cleaner (Alconox, 1104-1) and sonicated the coverslips for 20 min. After rinsing with deionized water, the coverslips were sonicated in acetone for 15 min and then sonicated again in 1 M NaOH or KOH for 20 min. Finally, we rinsed the coverslips with deionized water, followed by sonication 3 times for at least 5 min each time. The washed coverslips were stored in 95-100 % ethanol at 4 ℃.

The Hessian-SIM setup. The schematic illustration of the system is based on a commercial inverted fluorescence microscope (IX83, Olympus) equipped with a TIRF objective (Apo N 100× /1.7 HI Oil, Olympus), a multiband dichroic mirror (DM, ZT405/488/561/640-phase R; Chroma), a 488-nm laser (Sapphire 488LP-200), and a 561-nm laser (Sapphire 561LP-200, Coherent). Acoustic optical tunable filters (AOTF, AA Opto-Electronic, France) were used to combine, switch, and adjust illumination power of the lasers. A collimating lens (focal length: 10 mm, Lightpath) was used to couple the lasers to a polarization-maintaining single-mode fiber (QPMJ-3AF3S, Oz Optics). The output lasers were then collimated by an objective lens (CFI Plan

Apochromat Lambda 2 × N.A. 0.10, Nikon), and diffracted by the pure phase grating that consisted a polarizing beam splitter (PBS), a half wave plate and the SLM (3DM-SXGA, ForthDD). The diffraction beams were then focused by another achromatic lens (AC508-250, Thorlabs) onto the intermediate pupil plane, where a carefully designed stop mask was placed to block the zero-order beam and other stray light and to permit passage of ± 1 order beam pairs only. To maximally modulate the illumination pattern while eliminating the switching time between different excitation polarizations, a home mode polarization rotator placed after the stop mask [4]. Next, the light passed another lens (AC254-125, Thorlabs) and a tube lens (ITL200, Thorlabs) to focus on the back focal plane of the objective lens, which were interfered at the image plane after passing the objective lens. Emitted fluorescence collected by the same objective passed through a dichroic mirror (DM), an emission filter and another tube lens. Finally, the emitted fluorescence was split by an image splitter (W-VIEW GEMINI, Hamamatsu, Japan) before being captured by a sCMOS (Flash 4.0 V3, Hamamatsu, Japan).

Planarian care and cell transplantation. Asexual *Schmidtea mediterranea* clone CIW4 was starved for 7~10 days at 20 ℃ before each experiment. Animals exposed to 60 Gy of X-rays (Rad Source Technologies, RS2000 pro) were used as transplant hosts. SirNeoblasts stained with MitoTracker Red CMXRos or PK Mito Red were sorted with a BD Fusion flow cytometer and transplanted into irradiated hosts as previously described with minor modifications [5, 6]. Planarians were amputated post-pharyngeally and tail fragments were macerated in CMF + 1% bovine serum albumin for 20 min. Cell suspensions were filtered through 70 μm cell strainers (Biologix, 15-1070) and centrifuged at 290 ×g for 10 min at 4 ℃. Cell pellets were re-suspended in IPM + 10% Fetal Bovine Serum (FBS, Cellmax, SA211.02) and cells were stained with SiR-DNA (1 μM, Cytoskeleton Inc., CY-SC007) for 90 mins, Mito dye (MitoTracker Red CMXRox, 0.2 μg/ml, Thermo Fisher Technologies, M7512, or PK Mito Red, 0.2 μM) for 45 min, and CellTracker Green CMFDA Dye (2.5 μg/ml, Thermo Fisher Technologies, C7025) for 10 min at room temperature in dark. After staining, cell suspensions were centrifuged at 290 ×g for 10 min at 4 ℃. Cell pellets were re-suspended in IPM + 10% FBS and stained with DAPI (1 μg/ml, Thermo Fisher Technologies, D3571) for flow cytometry. Sorted cells were cultured in KnockOut DMEM containing 5% FBS (Cellmax, SA211.02) and 1 × Penicillin-Streptomycin (Thermo Fisher Technologies, 15070063). Approximately 1 μL of cell suspension (~ 5,000 cells/μL) was injected into the post-pharyngeal midline of asexual CIW4 hosts at 0.9 ~1.0 psi (Eppendorf FemtoJet) using a borosilicated glass microcapillary (Sutter Instrument Co., B100-75-15). Planarian hosts were fixed for whole mount in situ hybridization at 7 days post transplantation.

Whole mount in situ hybridization and microscopy. Whole-mount in situ hybridization (WISH) was carried out as previously described [7-10]. Colormetric WISH images were taken with a Zeiss V16 stereoscope. Fluorescence WISH images were taken with Nikon C2 confocal microscope. Image J, Adobe Photoshop and Illustrator were used for image and figure preparation. *Smedwi-1+* cell number was manually counted.

Cell in situ hybridization and viability measurement. In situ hybridization on cultured cells was performed as previously reported [6]. For viability measurement, cultured cells were resuspended and stained with DAPI. Quantitative examination was performed using a Moflo flow cytometer and Flowjo v10 was used for data analyses and figure preparation.

Chemical synthesis

General information. Unless otherwise mentioned, all reactions were carried out under a nitrogen atmosphere with dry solvents under anhydrous conditions. Anhydrous dichloromethane was distilled from calcium hydride, toluene was distilled from sodium. Reagents were purchased at the highest commercial quality and used without further purification, unless otherwise stated.

Reactions were monitored by Thin Layer Chromatography on plates (GF254) (Yantai Chemicals) using UV light as visualizing agent and an ethanolic solution of phosphomolybdic acid and cerium sulfate, and heat as developing agents or by LC/MS (4.6 mm × 150 mm 5 µm C18 column; 5 µL injection; 10–95% or 50–95% CH_3CN/H_2O, linear gradient, with constant 0.1% v/v TFA additive; 20 min run; 1 mL/min flow; ESI; positive ion mode; UV detection at 254 nm) . If not specially mentioned, flash column chromatography uses silica gel (200-300 mesh, Tsingtao Haiyang Chemicals).

NMR spectra were recorded on Bruker Advance 400 (^{1}H 400 MHz) and are calibrated using residual undeuterated solvent (Chloroform-*d* at 7.26 ppm ^{1}H NMR; Methanol-d_4 at 3.31 ppm ^{1}H NMR). The following abbreviations were used to explain the multiplicities: s = singlet, d = doublet, t = triplet, q = quartet, m = multiplet, br = broad. High resolution mass spectrometric data were obtained using Acquity I class UPLC synapt G2-SI using ESI (electrospray ionization).

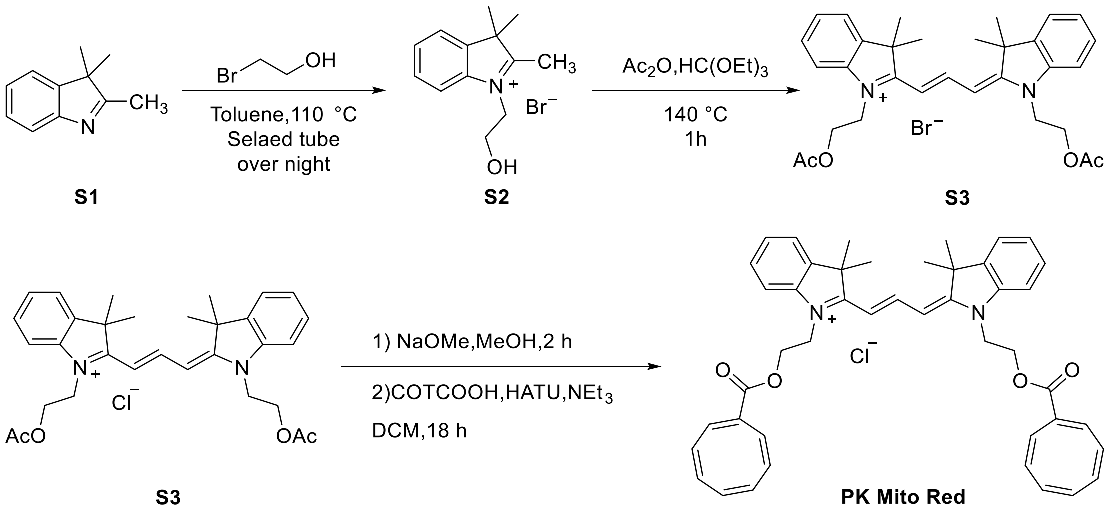

Supplementary Scheme 1 Synthetic route of PK Mito Red (compound **4**)

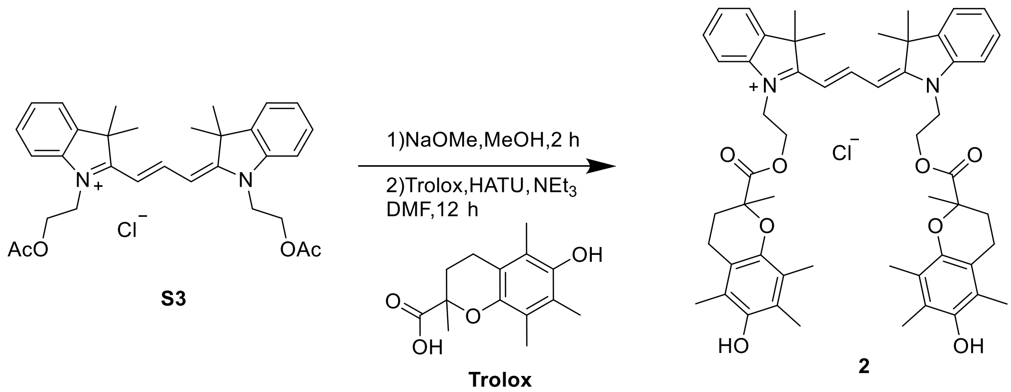

Supplementary Scheme 2 Synthetic route of compound **2**

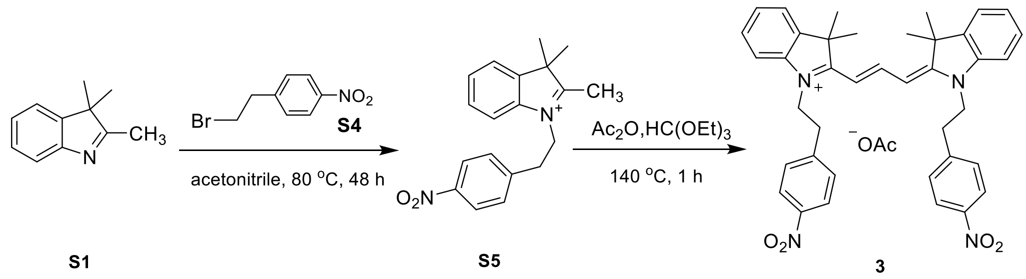

Supplementary Scheme 3 Synthetic route of compound **3**

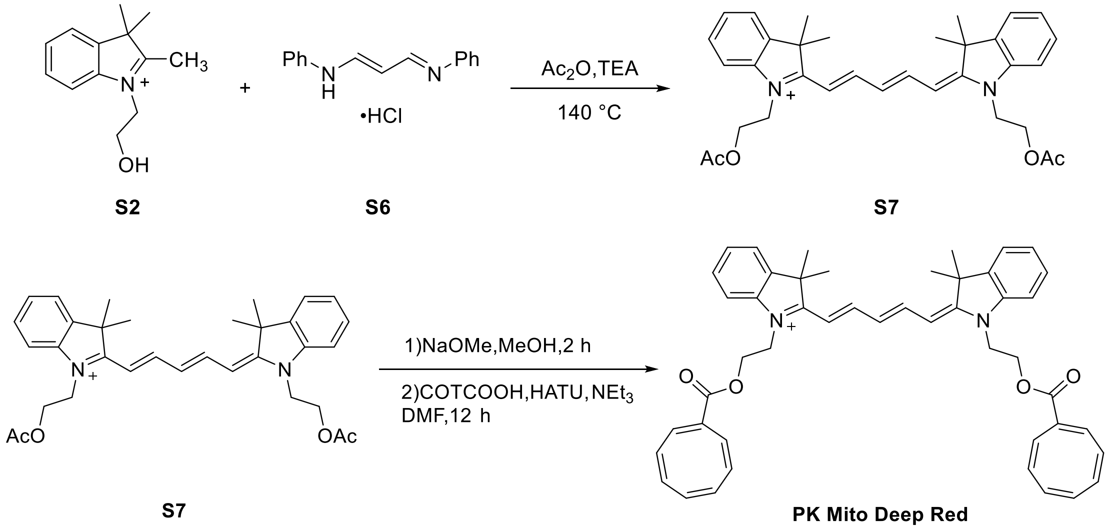

Supplementary Scheme 4 Synthetic route of PK Mito Deep Red (**5**)

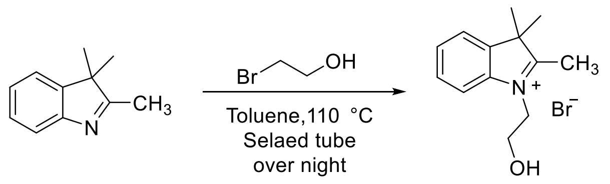

Compound S2: 2-Bromoethanol (706 mg, 0.4 mL, 5.65 mmol, 3.0 eq) was added to 2,3,3-trimethylindolenine (Compound **S1**; 300 mg, 1.88 mmol) solution in 5 mL toluene and the mixture was allowed to stirred at 110 ℃ over night in a sealed tube. After cooling to room temperature, the precipitated material was isolated by vacuum filtration and washed with cold Et_2O (2 × 20 mL) to obtain product **S2** (246 mg, 0.87 mmol, 46%) as a light pink salt.

^1H NMR (400 MHz, Methanol-d_4) δ 7.90 – 7.82 (m, 1H), 7.82 – 7.74 (m, 1H), 7.70 – 7.59 (m, 2H), 4.70 – 4.63 (t, *J* = 5.1 Hz, 2H,), 4.08 – 4.01 (t, *J* = 5.1 Hz, 2H), 1.62 (s, 6H).

The NMR is in accordance with published result, see reference [11].

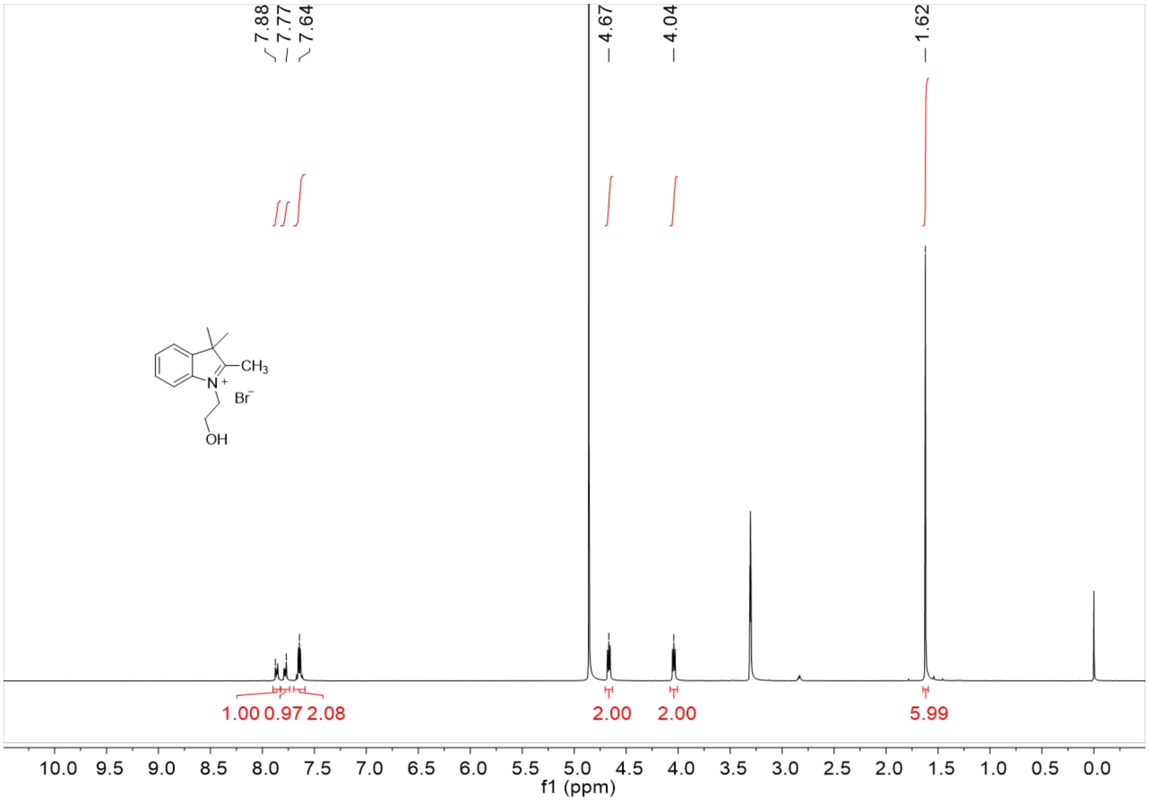

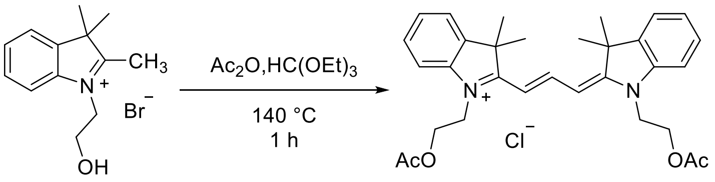

S2 S3

Compound S3: To a solution of compound **S2** (200 mg, 0.60 mmol) in 5 mL acetic anhydride was added triethyl orthoformate (133 mg, 0.9 mmol, 1.5 eq). The mixture was stirred at 140 ℃ for 1 hour. After cooling to room temperature, the mixture was poured into 20 mL brine. Dichloromethane (20 mL) was added and the organic layer was separated and the aqueous layer was extracted with 2 × 30 mL of dichloromethane. The extracts were combined, washed with 20 mL brine, dried with Na_2SO_4, filtered and concentrated. The residue was purified by flash chromatography on silica gel (1-10% MeOH/DCM) to afford 180 mg (0.29 mmol, 95%) of **S3** as a deep pink oil.

^{1}H NMR (400 MHz, Methanol-*d*$_4$) δ 8.60 (t, *J* = 13.4 Hz, 1H), 7.58 – 7.51 (m, 2H), 7.45 (ddd, *J* = 8.3, 7.1, 1.2 Hz, 2H), 7.44 – 7.37 (m, 2H), 7.32 (td, *J* = 7.3, 1.2 Hz, 2H), 6.60 (d, *J* = 13.4 Hz, 2H), 4.60 – 4.54 (m, 4H), 4.54 – 4.46 (m, 4H), 1.83 (s, 6H), 1.77 (s, 12H).

^{13}C NMR (101 MHz, Methanol-*d*$_4$) δ 175.63, 170.84, 151.24, 142.20, 140.66, 128.46, 125.49, 122.14, 111.21, 102.84, 60.23, 49.39, 43.22, 26.81, 19.16.

HRMS (ESI) calcd for $C_{31}H_{37}N_2O_4^+$[M$^+$] 501.2748, found 501.2753.

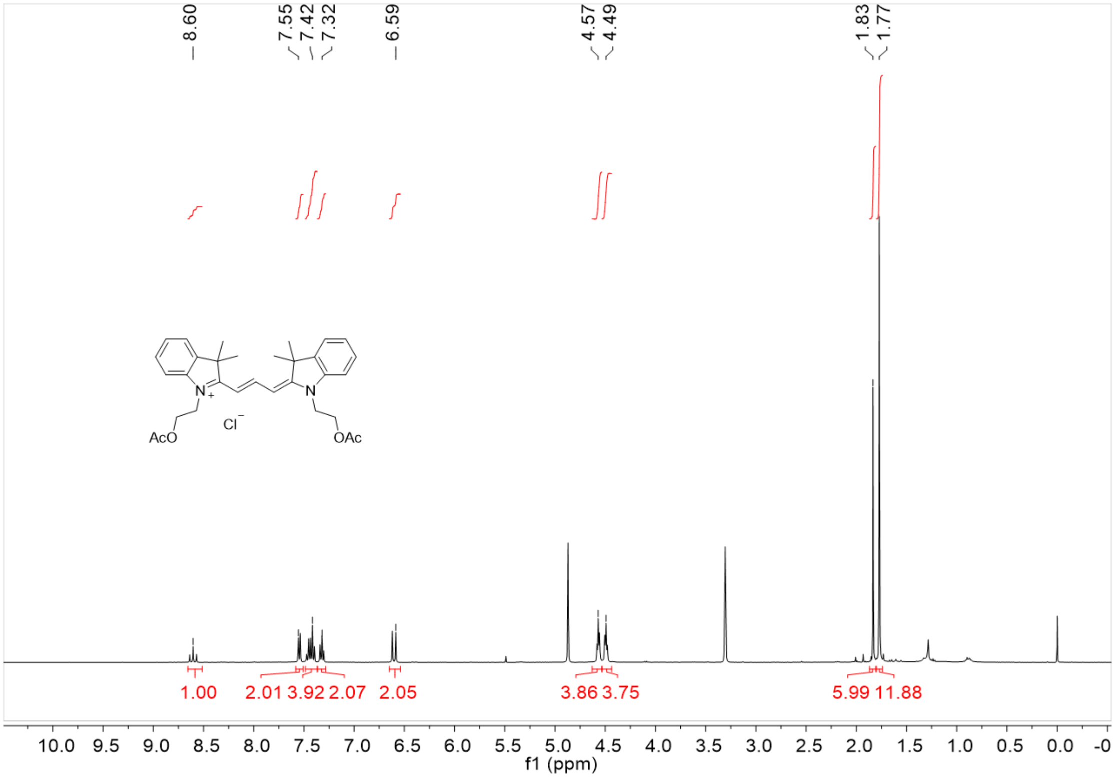

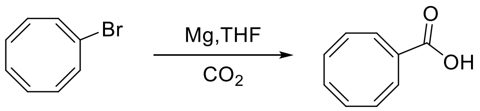

Cyclooctatetraenecarboxylic acid (COTCOOH): A molar excess of magnesium ribbon (2.0 g, 83 mmol), polished with emery paper, was cut directly into a 50 mL round bottom flask purged with argon gas. COTBr (2.0 g, 11.6 mmol) was dissolved in THF, then the solution was added dropwisely to the flask containing the Mg ribbon. The heterogeneous mixture was stirred at 0 ℃ under argon for approximately 1 h and then for an additional 4 h at room temperature until a dark blue-green colored solution was generated indicating formation of the Grignard reagent, COTMgBr. The resulting solution was cooled to −78 °C, followed by the addition of excess solid dry ice (CO_2). The reaction was quenched with 20 mL water and acidified to pH = 2 with 1 M HCl. The resulting carboxylic acid was extracted from the aqueous solution using pentane (3 × 30 mL) then purified by flash chromatography on silica gel (20% EA/PE) to afford 1.05g (7.4 mmol 62%) cyclooctatetraenecarboxylic acid (COTCOOH).

^{1}H NMR (400 MHz, Chloroform-*d*) 7.16 (s, 1H), 6.06 – 5.96 (m, 1H), 5.93 (d, *J* = 10.9 Hz, 3H), 5.90 – 5.84 (m, 1H), 5.81 (s, 1H).

The NMR is in accordance with published result, see reference [12].

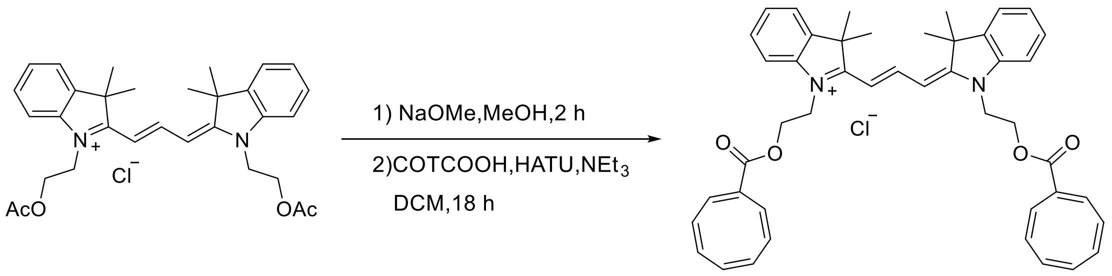

S3 **PK Mito Red**

PK Mito Red: Compound **S3** (30 mg, 0.047 mmol) was dissolved in 2 mL MeOH and then NaOMe (15.4 mg, 0.2 mmol, 6.0 eq) was added. The mixture was stirred at room temperature for 2 h. Solid was removed by filtration and then the solvent was removed *in vacuo* to give 18 mg (0.033 mmol, 70%) diol as a deep pink solid. Cyclooctatetraenecarboxylic acid (10 mg, 0.067 mmol, 2.0 eq) was dissolved in 2 mL DCM. Hexafluorophosphate Azabenzotriazole Tetramethyl Uronium (HATU, 50 mg, 0.134 mmol, 4.0 eq) and triethylamine (18 μL, 0.134 mmol, 4.0 eq) was subsequently added at 0 ℃. The mixture was stirred at 0 ℃ for 10 mins. Then a DCM solution (3 mL) of diol was added to this mixture at 0 ℃. The resulting mixture was stirred at 0 ℃ for another 15 min before warmed to room temperature and stirred for another 18 hours. The mixture was concentrated under reduced pressure, and the residue was diluted with dichloromethane, washed with 10 mL saturated aqueous sodium bicarbonate solution and 10 mL saturated brine, and dried over anhydrous magnesium sulfate. The solution was subsequently concentrated *in vacuo*, and then purified by flash chromatography on silica gel (1-10% MeOH/DCM) to afford 12 mg (0.018 mmol, 53%) of **PK Mito Red** as a deep pink solid.

^{1}H NMR (400 MHz, Methanol-*d*$_4$) δ 8.59 (t, *J* = 13.4 Hz, 1H), 7.56 (d, *J* = 7.5 Hz, 2H), 7.51 – 7.38 (m, 4H), 7.35 (t, *J* = 7.4 Hz, 2H), 6.88 (s, 1H), 6.53 (d, *J* = 13.4 Hz, 2H), 5.95 – 5.66 (m, 12H), 4.65 (t, *J* = 5.0 Hz, 4H), 4.54 (t, *J* = 5.2 Hz, 4H), 1.78 (s, 12H).

^{13}C NMR (101 MHz, Chloroform-*d*) δ 174.60, 165.25, 151.16, 143.54, 142.44, 140.28, 133.94, 132.94, 132.62, 132.02, 131.35, 130.04, 129.30, 128.88, 125.45, 122.02, 111.30, 104.52, 60.96, 49.15, 43.37, 28.09.

HRMS (ESI) calcd for $C_{45}H_{45}N_2O_4^+$ [M^+] 677.3374, found 677.3377.

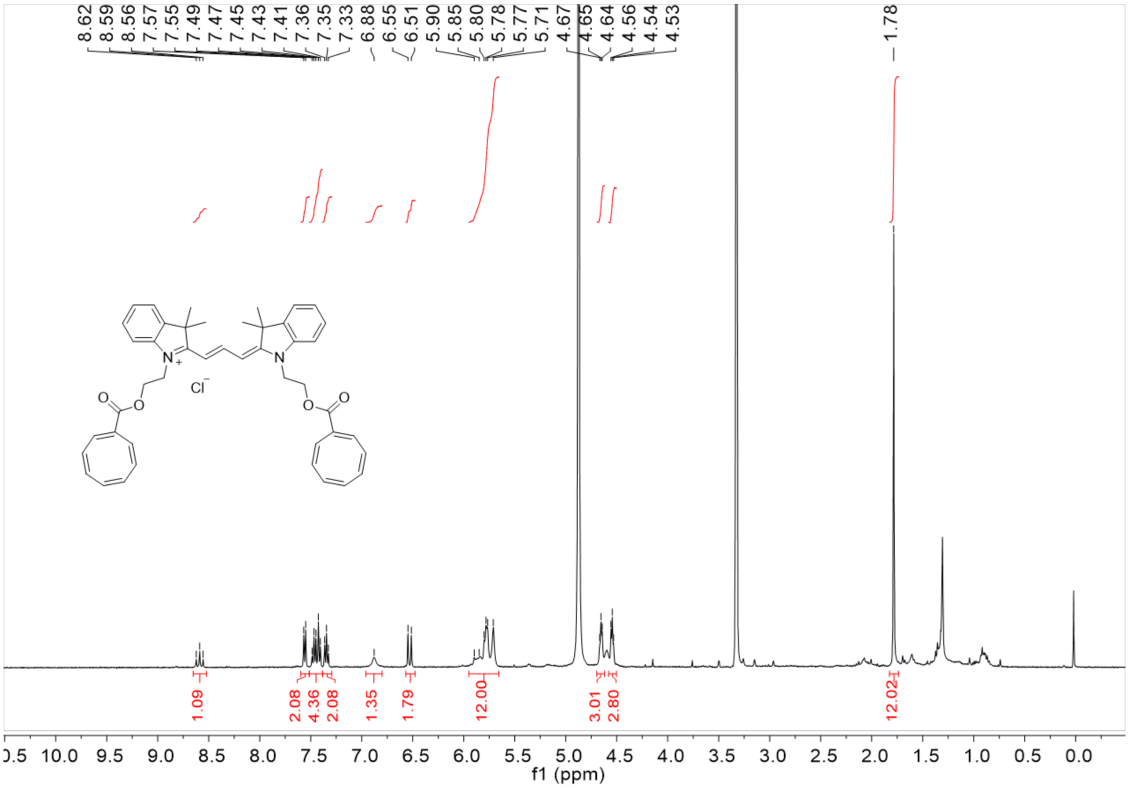
8.62 8.59 8.56 7.57 7.55 7.49 7.47 7.45 7.43 7.41 7.36 7.35 7.33 6.88 6.55 6.51 5.90 5.85 5.80 5.78 5.77 5.71 4.67 4.65 4.64 4.56 4.54 4.53
1.78
1.09 2.08 4.36 2.08 1.35 1.79 12.00 3.01 2.80 12.02
Cl⁻
f1 (ppm)

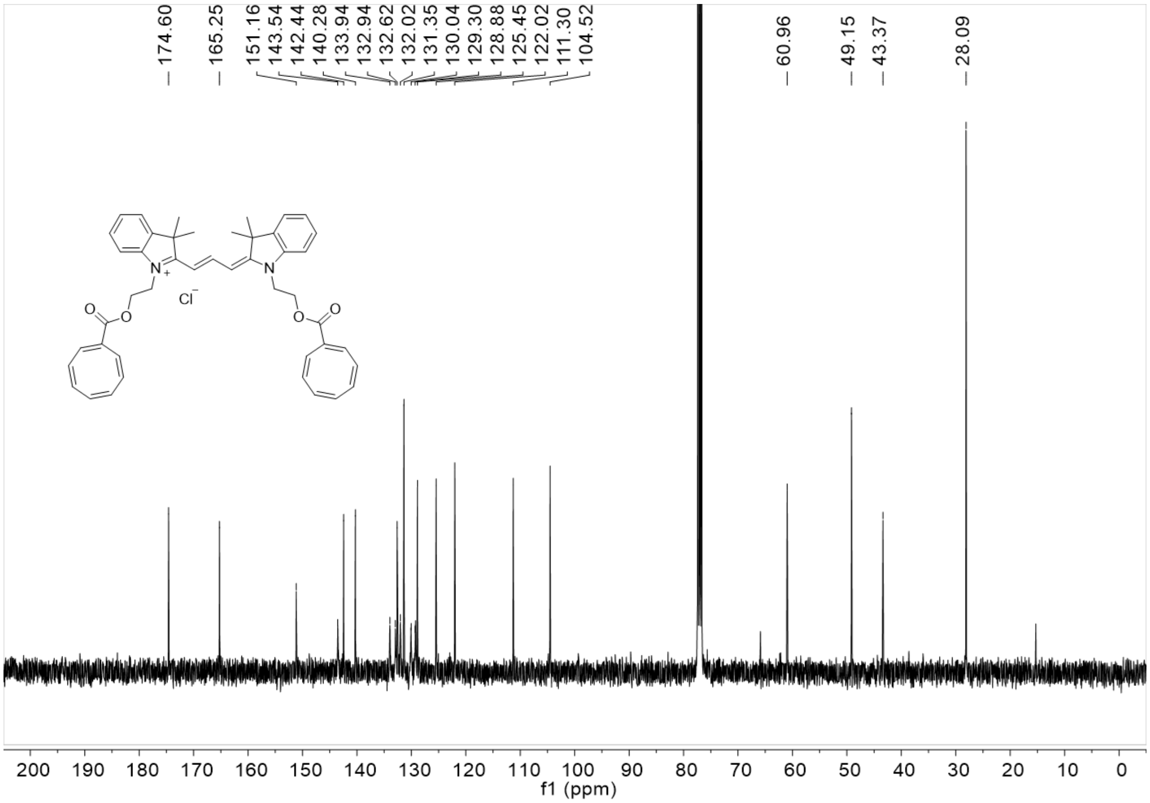
174.60
165.25
151.16 143.54 142.44 140.28 133.94 132.94 132.62 132.02 131.35 130.04 129.30 128.88 125.45 122.02 111.30 104.52
60.96
49.15
43.37
28.09
Cl⁻
f1 (ppm)

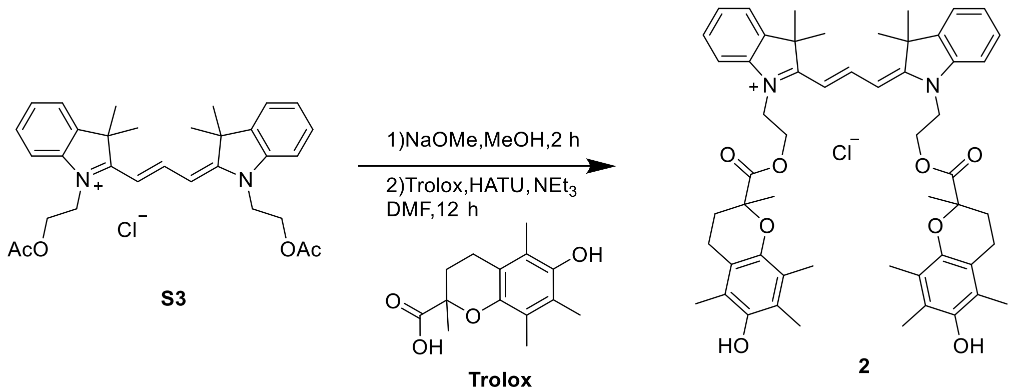

Compound 2: Compound S3 (30 mg, 0.047 mmol) was dissolved in 2 mL MeOH and then NaOMe (15.4 mg, 0.2 mmol, 6.0 eq) was added. The mixture was stirred at room temperature for 2 h. Solid was removed by filtration and then the solvent was removed in vacuo to give 18 mg (0.033 mmol, 70%) diol as a deep pink solid. Trolox (18 mg, 0.072 mmol, 3.0 eq) was dissolved in 2 mL DCM. Hexafluorophosphate Azabenzotriazole Tetramethyl Uronium (HATU, 27.3 mg, 0.072mmol, 3.0 eq) and triethylamine (15 μL, 0.12 mmol, 5.0 eq) was subsequently added at 0 ℃. The mixture was stirred at 0 ℃ for 10 mins. Then a DCM solution (3 mL) of diol (10 mg, 0.024 mmol, 1.0 eq) was added to this mixture at 0 ℃. The resulting mixture was stirred at 0 ℃ for another 15 min before warmed to 40 ℃ and stirred for another 12 h. The mixture was concentrated under reduced pressure, and the residue was diluted with dichloromethane, washed with 10 mL saturated aqueous sodium bicarbonate solution and 10 mL saturated brine, and dried over anhydrous magnesium sulfate. The solution was subsequently concentrated in vacuo, and then purified by flash chromatography on silica gel (4% MeOH/DCM) to afford 15 mg (0.018 mmol, 68%) of compound **2** as a red solid.

^{1}H NMR (400 MHz, Methanol-*d*$_4$) δ 8.56 (t, *J* = 13.4 Hz,1H), 8.00 (s, 1H), 7.57 (dd, *J* = 7.5, 1.2 Hz, 2H), 7.49 – 7.40 (m, 2H), 7.40 (d, *J* = 8.0 Hz, 2H), 7.35 (td, *J* = 7.3, 1.2 Hz, 2H), 6.49 (d, *J* = 13.4 Hz, 1H), 4.68 – 4.57 (m, 1H), 4.53 – 4.40 (m, 3H), 3.01 (s, 6H), 2.88 (d, *J* = 0.7 Hz, 6H), 2.83 (s, 6H), 2.25 – 2.10 (m, 4H), 2.13 – 2.00 (m, 4H), 1.94 (s, 6H), 1.80 – 1.73 (m, 12H).

MS (ESI) calcd for $C_{45}H_{45}N_2O_4^+$ [M$^+$] 881.47, found 881.86.

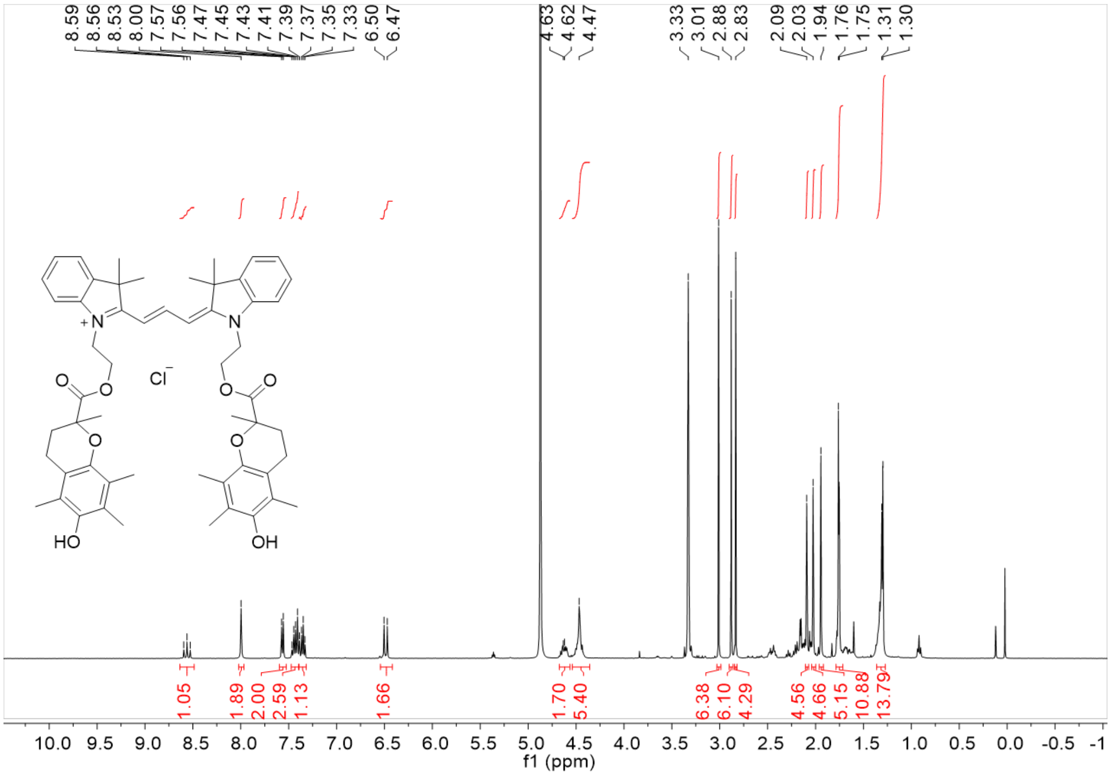

8.59
8.56
8.53
8.00
7.57
7.56
7.47
7.45
7.43
7.41
7.39
7.37
7.35
7.33
6.50
6.47
4.63
4.62
4.47
3.33
3.01
2.88
2.83
2.09
2.03
1.94
1.76
1.75
1.31
1.30
Cl⁻
HO
OH
1.05
1.89
2.00
2.59
1.13
1.66
1.70
5.40
6.38
6.10
4.29
4.56
4.66
5.15
10.88
13.79
10.0 9.5 9.0 8.5 8.0 7.5 7.0 6.5 6.0 5.5 5.0 4.5 4.0 3.5 3.0 2.5 2.0 1.5 1.0 0.5 0.0 -0.5 -1
f1 (ppm)

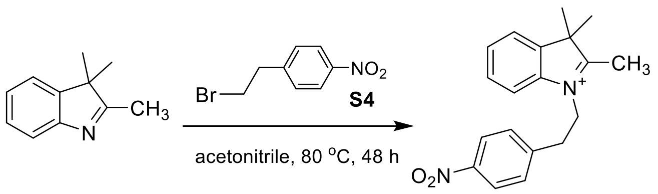

S1 S5

Compound S5: A mixture of compound **S1** (1.00 g, 6.28 mmol, 1.00 eq) and compound **S4** (2.89 g, 12.7 mmol, 2.00 eq) in acetonitrile (7.00 mL) was heated to 80 ℃ and stirred at this temperature for 48 h. The mixture was cooled to room temperature in an ice bath and solid was collected by filtration, the solid was washed with ethyl acetate (10.0 mL, 5.00 mL) to give the compound **S5** (1.00 g, 3.23 mmol, 51.5% yield) as a white solid.

^{1}H NMR (400 MHz, DMSO-d_6) δ 8.15 (d, J = 8.4 Hz, 2H), 7.96 - 7.98 (m, 1H), 7.81 - 7.83 (m, 1H), 7.57 - 7.62 (m, 4H), 4.80 (t, J = 7.2 Hz, 2H), 3.37 (t, J = 7.2 Hz, 2H), 2.57 (s, 3H), 1.45 (s, 6H)

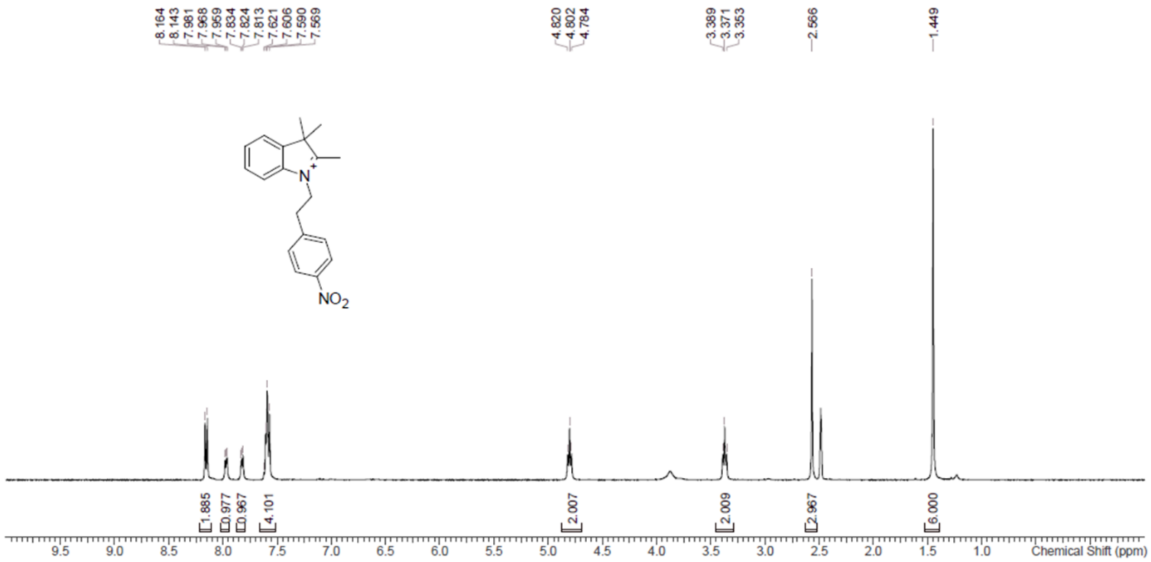

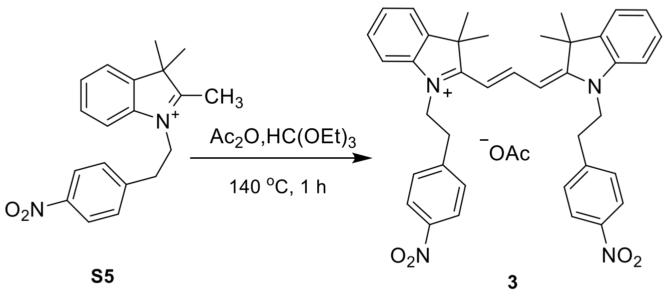

S5 **3**

Compound 3: Compound **S5** (100 mg, 323 umol, 1.00 eq) and acetic anhydride (215 mg, 2.10 mmol, 197 uL, 6.50 eq) were added to a 50 mL flask, triethyl orthoformate (71.9 mg, 485 umol, 80.6 uL, 1.50 eq) was added dropwise into the reaction mixture, followed by heating to 140 ℃ and stirred at this temperature for 1 h. The reaction mixture was diluted with EtOAc (2.0 mL) when cooled to room temperature and the precipitate was collected by filtration to give a brown solid, which was washed with EtOAc (2.0 mL x 2) to give compound **2** (50 mg, 72.7 umol, 45.0% yield, HOAC) as a brown solid.

^{1}H NMR (400 MHz, DMSO-d_6) δ 8.04 - 8.14 (m, 5H), 7.58 - 7.61 (m, 6H), 7.61 - 7.38 (m, 4H), 7.24 - 7.27 (m, 2H), 6.17 (d, J = 13.6 Hz, 2H), 4.46 (t, J = 7.2 Hz, 2H), 3.23 (t, J = 7.2 Hz, 4H), 1.89 (s, 3H), 1.58 (s, 12H)

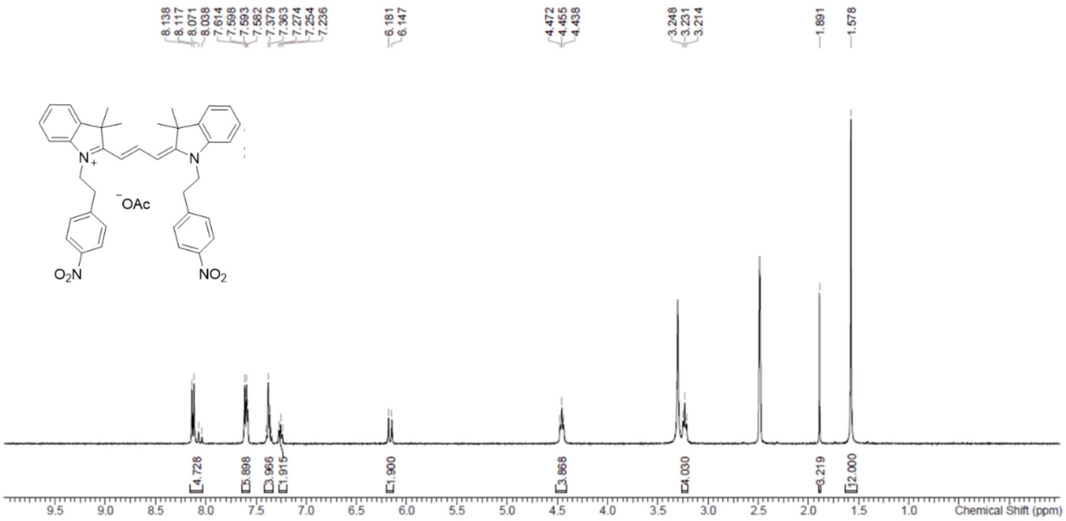

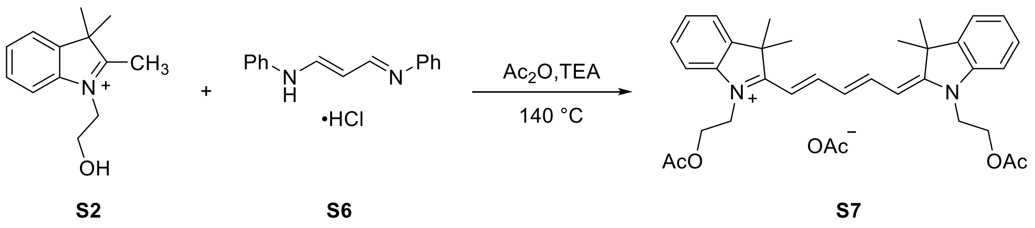

Compound S7: To a solution of compound **2** (100 mg, 0.30 mmol, 2.0 eq) and phenyl[3-phenylaminoprop-2-en-1-ylidene] ammonium chloride (39 mg, 0.15 mmol, 1.0 eq) in acetic anhydride was added sodium acetate (24.6 mg, 0.3 mmol, 2.0 eq). The mixture was heated to 110 ℃ for 1 h in a sealed tube. After cooling to room temperature, the mixture was poured into 20 mL brine. Dichloromethane (30 mL) was added and the organic layer was separated and the washed with brine, dried with Na_2SO_4, filtered and concentrated. The residue was purified by flash chromatography (1-10% MeOH/DCM) to give 91 mg (0.139 mmol, 93%) compound **S7**.

^{1}H NMR (400 MHz, Methanol-*d*$_4$) δ 8.32 (t, *J* = 13.0 Hz, 2H), 7.49 (dd, *J* = 7.5, 1.2 Hz, 2H), 7.42 (td, *J* = 7.7, 1.2 Hz, 2H), 7.34 (d, *J* = 7.9 Hz, 2H), 7.27 (td, *J* = 7.4, 1.0 Hz, 2H), 6.65 (t, *J* = 12.4 Hz, 1H), 6.39 (d, *J* = 13.7 Hz, 2H), 4.52 (t, *J* = 5.1 Hz, 4H), 4.42 (t, *J* = 5.1 Hz, 4H), 1.85 (s, 6H), 1.73 (s, 12H).

^{13}C NMR (101 MHz, Methanol-*d*$_4$) δ 174.30, 170.85, 154.67, 144.93, 142.28, 141.11, 128.23, 124.97, 122.03, 110.70, 103.40, 60.26, 49.30, 42.84, 26.43, 19.16.

HRMS (ESI) calcd for $C_{33}H_{39}N_2O_4^+$ [M$^+$] 527.2904, found 527.2915.

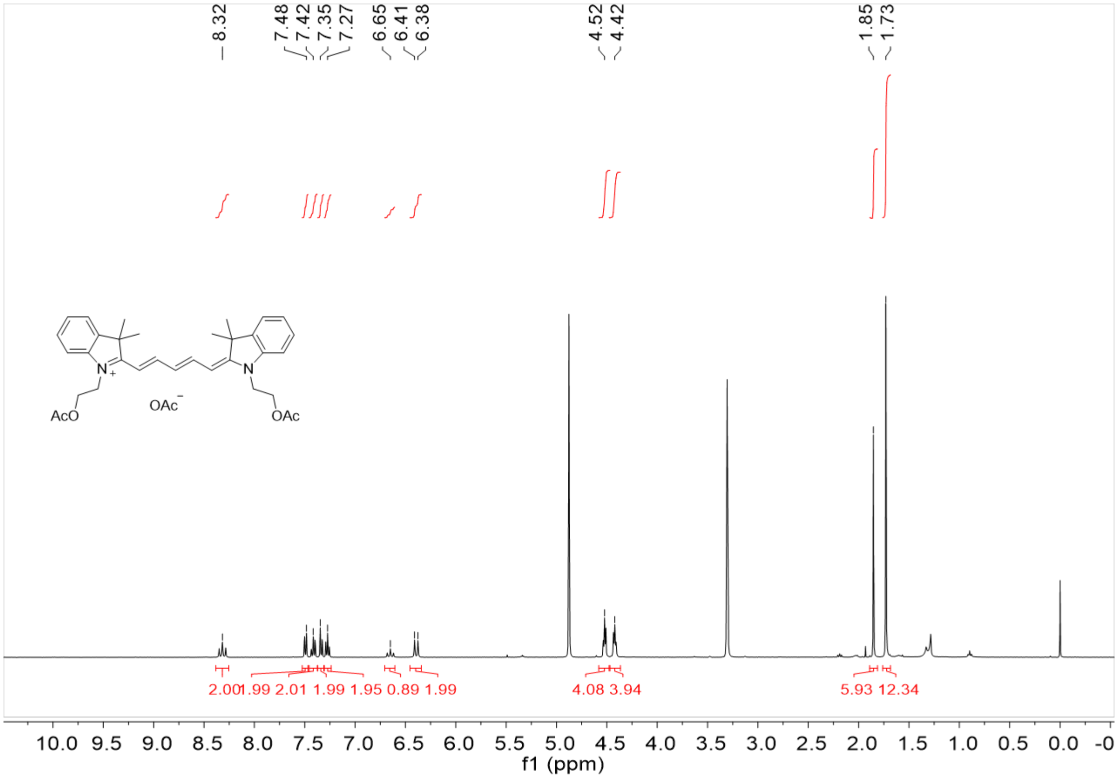

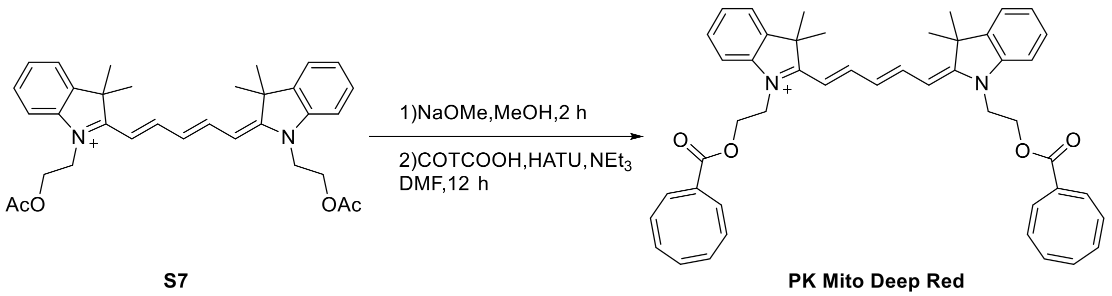

PK Mito Deep Red: Compound **S7** (91 mg, 0.139 mmol) was dissolved in 10 mL MeOH and then NaOMe (45 mg, 0.834 mmol, 6.0 eq) was added. The mixture was stirred at room temperature for 2 hours. Solid was removed by filtration and then the solvent was removed, then the diol was dissolved in 5 mL DCM. Cyclooctatetraenecarboxylic acid (13 mg, 0.082 mmol, 2.0 eq) was dissolved in 5 mL DMF. Then HATU (38 mg, 0.10mmol, 2.5eq) and triethylamine (25 μL, 0.16 mmol, 4.0 eq) was added to this solution at 0 ℃. The mixture was stirred at 0 ℃ for 10 min. Then the DCM solution of diol was added to this solution. The resulting mixture was stirred at 0 ℃ for another 15 min, then it was allowed to rise to room temperature and was stirred for another 18 hours. The reaction mixture was then diluted with dichloromethane (20 mL), washed with saturated aqueous sodium bicarbonate solution (20 mL) and saturated brine (20 mL), and dried over anhydrous magnesium sulfate. The solution was subsequently concentrated *in vacuo*, and then purified by flash chromatography on silica gel (1-10% MeOH/DCM) to afford 17 mg (0.02 mmol, 14%) of **PK Mito Deep Red** as a deep blue solid.

^{1}H NMR (400 MHz, Methanol-d_4) δ 8.30 (t, J = 13.1 Hz, 2H), 7.50 (d, J = 7.5 Hz, 2H), 7.43 (t, J = 7.7 Hz, 2H), 7.35 (d, J = 8.0 Hz, 2H), 7.29 (t, J = 7.4 Hz, 2H), 6.89 (s, 2H), 6.61 (t, J = 12.4 Hz, 1H), 6.39 (d, J = 13.7 Hz, 2H), 5.92 – 5.70 (m, 12H), 4.62 (t, J = 5.0 Hz, 4H), 4.49 (t, J = 5.1 Hz, 4H), 1.74 (s, 12H).

^{13}C NMR (101 MHz, Chloroform-d) δ 173.97, 165.30, 153.95, 143.97, 142.01, 141.09, 134.29, 133.38, 132.46, 132.03, 131.44, 129.94, 129.02, 128.59, 126.57, 125.37, 122.32, 60.61, 49.61, 43.01, 27.87.

HRMS (ESI) calcd for $C_{47}H_{47}N_2O_4^+$ [M^+] 703.3530, found 703.3520.

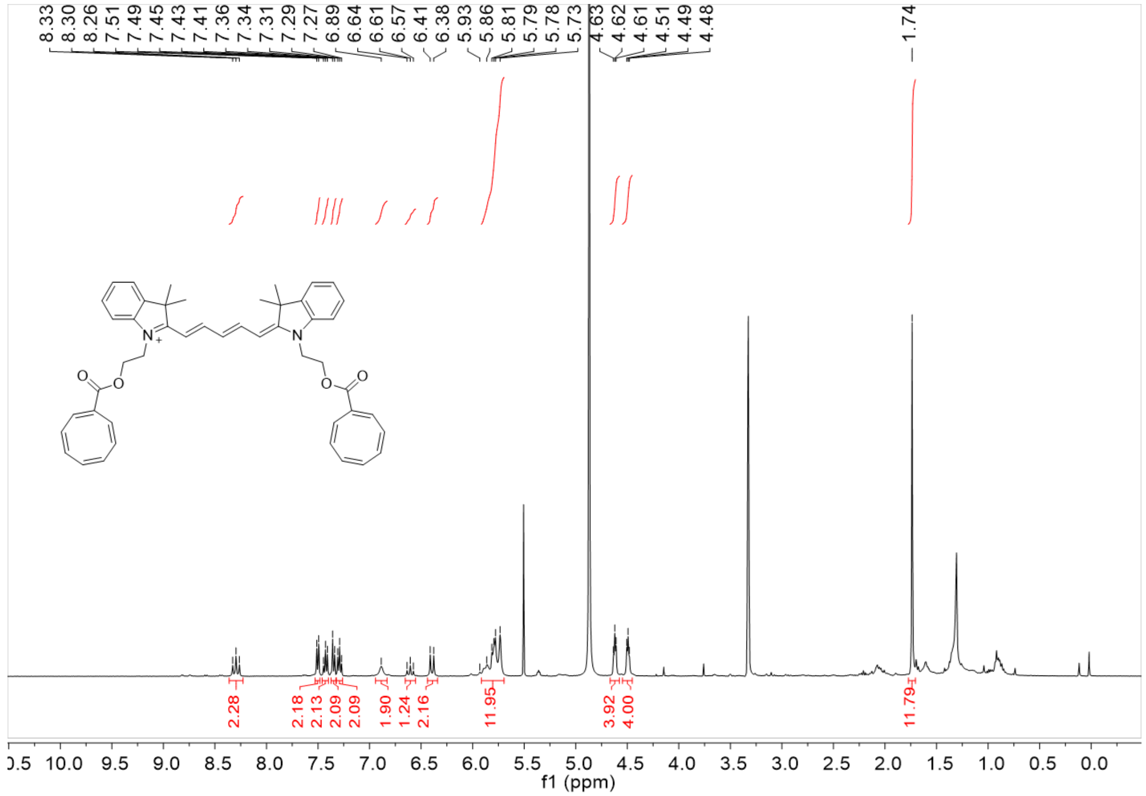
8.33
8.30
8.26
7.51
7.49
7.45
7.43
7.41
7.36
7.34
7.31
7.29
7.27
6.89
6.64
6.61
6.57
6.41
6.38
5.93
5.86
5.81
5.79
5.78
5.73
4.63
4.62
4.61
4.51
4.49
4.48
1.74
2.28
2.18
2.13
2.09
2.09
1.90
1.24
2.16
11.95
3.92
4.00
11.79
f1 (ppm)

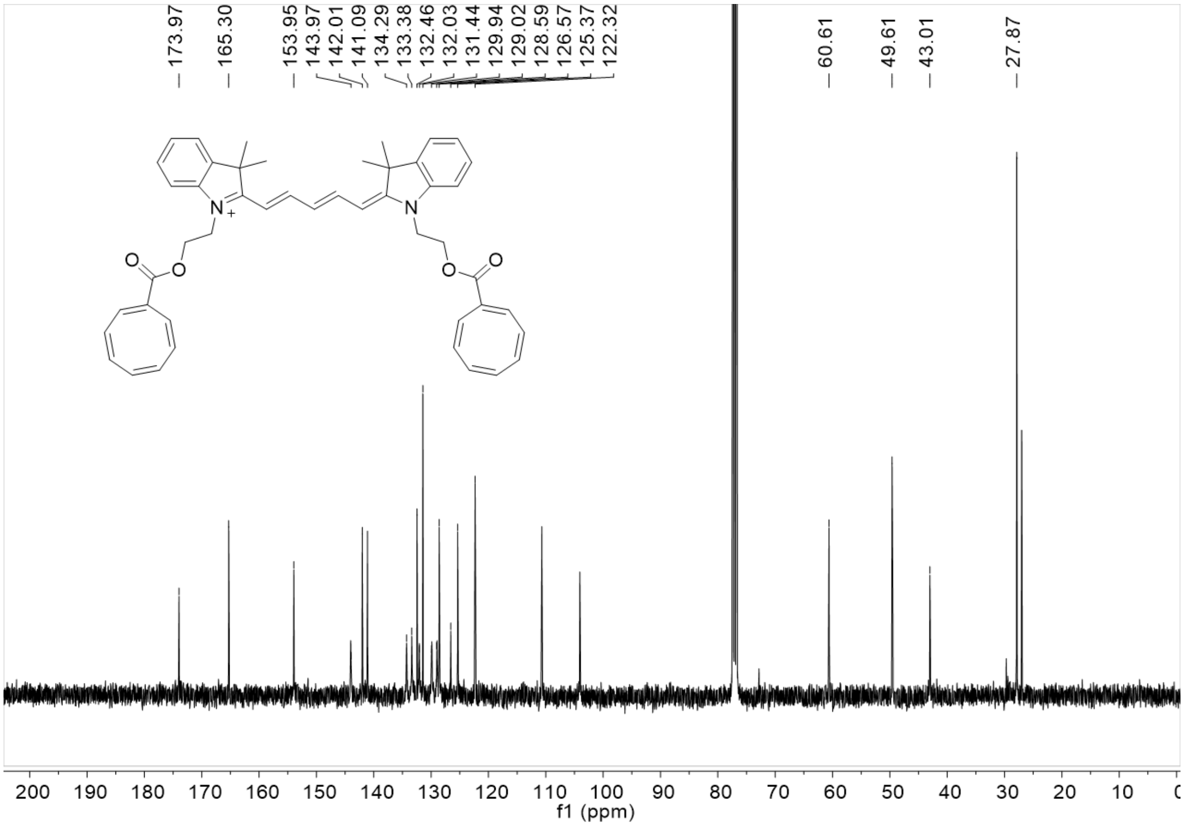
173.97
165.30
153.95
143.97
142.01
141.09
134.29
133.38
132.46
132.03
131.44
129.94
129.02
128.59
126.57
125.37
122.32
60.61
49.61
43.01
27.87
f1 (ppm)

References

1. V. Biju, M. Yamauchi and M. Ishikawa, *J. Photoch. Photobio A*, 2001, **140**, 237-241.
2. M. Poot, Y. Z. Zhang, J. A. Krämer, K. S. Wells, L. J. Jones, D. K. Hanzel, A. G. Lugade, V. L. Singer and R. P. Haugland, *J. Histochem. Cytochem.*, 1996, **44**, 1363-1372.
3. H. Cheng, W. J. Lederer and M. B. Cannell, *Science*, 1993, **262**, 740.
4. X. Huang, J. Fan, L. Li, H. Liu, R. Wu, Y. Wu, L. Wei, H. Mao, A. Lal, P. Xi, L. Tang, Y. Zhang, Y. Liu, S. Tan and L. Chen, *Nat. Biotechnol.*, 2018, **36**, 451-459.
5. D. E. Wagner, I. E. Wang and P. W. Reddien, *Science*, 2011, **332**, 811.
6. K. Lei, S. A. McKinney, E. J. Ross, H.-C. Lee and A. Sánchez Alvarado, *bioRxiv*, 2019, 573725.
7. B. J. Pearson, G. T. Eisenhoffer, K. A. Gurley, J. C. Rink, D. E. Miller and A. Sánchez Alvarado, *Dev. Dyn.*, 2009, **238**, 443-450.
8. R. S. King and P. A. Newmark, *BMC Dev. Biol.*, 2013, **13**, 8.
9. H. Thi-Kim Vu, J. C. Rink, S. A. McKinney, M. McClain, N. Lakshmanaperumal, R. Alexander and A. Sánchez Alvarado, *eLife*, 2015, **4**, e07405.
10. K. Lei, H. Thi-Kim Vu, Ryan D. Mohan, Sean A. McKinney, Chris W. Seidel, R. Alexander, K. Gotting, Jerry L. Workman and A. Sánchez Alvarado, *Dev. Cell*, 2016, **38**, 413-429.
11. S. Friedle and S. W. Thomas Iii, *Angew. Chem. Int. Ed.*, 2010, **49**, 7968-7971.
12. S. J. Peters and J. R. Klen, *J. Org. Chem.*, 2015, **80**, 5851-5858.
